# Characterization of antibiogram fingerprints in *Listeria monocytogenes* recovered from irrigation water and agricultural soil samples

**DOI:** 10.1371/journal.pone.0228956

**Published:** 2020-02-10

**Authors:** Chidozie Declan Iwu, Anthony Ifeanyi Okoh

**Affiliations:** 1 SAMRC Microbial Water Quality Monitoring Centre, University of Fort Hare, Alice, South Africa; 2 Applied and Environmental Microbiology Research Group, Department of Biochemistry and Microbiology, University of Fort Hare, Alice, South Africa; MAHSA University, MALAYSIA

## Abstract

*Listeria monocytogenes* (*L*. *monocytogenes*) is a foodborne pathogen and the etiologic agent of listeriosis, which can be disseminated within the agricultural environment particularly soil and irrigation water, contaminate farm produce and cause high mortality and morbidity among vulnerable individuals. This study assessed the incidence and antibiogram of *L*. *monocytogenes* recovered from irrigation water and agricultural soil samples collected from Chris Hani and Amathole District Municipalities (DMs) in Eastern Cape Province, South Africa. The distribution of presumptive *L*. *monocytogenes* in irrigation water and agricultural soil samples was done using the standard plate count method, while polymerase chain reaction (PCR) was used to identify the isolates. The confirmed isolates were screened for 9 key virulence markers using PCR after which they were subjected to antibiotic susceptibility testing against 18 antibiotics used for the alleviation of listeriosis using the disk diffusion method. Relevant putative antibiotic resistance genes in the resistant variants were screened for using PCR. The distribution of *L*. *monocytogenes* in irrigation water samples was statistically significant (*P* ≤ 0.05) and ranged from log_10_ 1.00 CFU/100ml to log_10_ 3.75 CFU/100 ml. In agricultural soil samples, the distribution ranged significantly (*P* ≤ 0.05) from log_10_ 2.10 CFU/g to log_10_ 3.51 CFU/g. Of the 117 presumptive *L*. *monocytogenes* recovered from irrigation water samples and 183 presumptive *L*. *monocytogenes* isolated from agricultural soil samples, 8 (6.8%) and 12 (6.6%) isolates were confirmed respectively. Nine virulence genes including *inlA*, *inlB*, *inlC*, *inlJ*, *actA*, *hlyA*, *plcA*, *plc*B, and *iap* were detected in all the isolates. The proportion of the isolates exhibiting phenotypic resistance against the test antimicrobials followed the order: tetracycline (90%), doxycycline (85%), cefotaxime (80%), penicillin (80%), chloramphenicol (70%), linezolid (65%), erythromycin (60%) and trimethoprim/sulfamethoxazole (55%). The isolates exhibited multiple antibiotic resistance against 3 or more antibiotics and the MAR indices of all the multidrug isolates were ≥0.2. The isolates harboured antibiotic resistance genes including *tet*A, *tet*B, *tet*C, *sul*I, *sul*II, *aadA*, *aac(3)-IIa* and ESBLs including *bla*_TEM_, *bla*_CTX-M_ group 9, *bla*_VEB_ *as* well as *AmpC*. None of the isolates harboured the carbapenemases. We conclude that irrigation water and agricultural soil collected from Chris Hani and Amathole District Municipalities (DMs) in Eastern Cape Province of South Africa are reservoirs and potential transmission routes of multidrug-resistant *L*. *monocytogenes* to the food web and consequently threat to public health.

## Introduction

*L*. *monocytogenes* is a foodborne, Gram-positive pathogen belonging to the genus *Listeria* and responsible for the sporadic and epidemic outbreak of the disease listeriosis. Listeriosis mostly affects the vulnerable people including the immunocompromised, pregnant women, paediatrics as well as geriatrics [1]. Following the consumption of infectious dose of *L*. *monocytogenes*, listeriosis can manifest in twofold- either as non-invasive gastrointestinal listeriosis which is observed among the immunocompetent people or the invasive listeriosis which is observed mostly among the immunocompromised, making them to come down with serious health issues like gastroenteritis, encephalitis, meningitis, septicemia, stillbirths and abortion [2]. Unlike other foodborne diseases, the frequency of occurrence of listeriosis is fairly low and this is owed to the erraticism in the pathogenicity of various strains of *L*. *monocytogenes* [3]. However, it has a high fatality rate ranging between 20–40%, especially among the “at-risk group” [4]. A 42% fatality rate was recently reported in the NICD (National Institute for Communicable disease) final outcome data on the listeriosis outbreak in South Africa targeting mostly infants and pregnant women [5].

The Food and Drug Administration (FDA) reported that about 2500 individuals in the USA suffer listeriosis every year [6]. Nineteen percent of all the deaths resulting from ingesting contaminated foods in the USA are caused by *L*. *monocytogenes* [7]. Recently, sporadic listeriosis incidence has been documented in some European countries such as Finland, France, United Kingdom, Denmark, and Belgium [2]. In South Africa, NICD on the 12^th^ of January, 2018 reported the largest listeriosis outbreak the country has ever experienced with over 748 laboratory-confirmed cases [5]. In the Eastern Cape Province, 50 cases out of which 10 deaths were reported. This is related to the high virulence nature of *L*. *monocytogenes* which is usually determined by numerous molecular factors which are involved in the various phases of the infection. Usually, at the beginning of infection, the *inlA*, *inlB*, *inlF* and *inlJ* genes are involved with adhesion and invasion [8]. The *hly*, *mpl*, *prfA*, *actA*, and *plcB* genes promote the growth and spread of the pathogen within and between host cells respectively [8]. The *hyl* gene mediates the discharge of the bacterial cells into the cytoplasm of the host. The *plcA* and *plcB* promote the escape of the bacteria from bound vacuoles. The surface protein actin A (*actA*) mediates the cell to cell dissemination of the bacteria [8].

*L*. *monocytogenes* are ubiquitous in nature often occurring in environments like faeces, manure, soil, aquatic sources and other channels through which the pathogen can find its way into the food web [9]. *L*. *monocytogenes* can persist under harsh temperature, high hydrostatic pressure, pH, extreme energy, oxidative stress, and high salt concentration [10], no wonder they are able to survive those adverse conditions employed to control foodborne pathogens. *L*. *monocytogenes* was initially considered as a human-related foodborne pathogen in 1981 after the contaminated coleslaw linked outbreak of listeriosis in Canada [11]. Since then, it is believed that over 99% of listeriosis in humans results from the ingestion of contaminated foodstuffs, especially the ready-to-eat (RTE) foods including meat products, pasteurized and unpasteurized dairy products, seafood, fruits, vegetables, and other fresh produce like cabbage, corn, carrots, cucumber, parsley [2,5]. *L*. *monocytogenes* present in animal and human faeces are transferred to food crops through the discharge of sewage materials and manure to agricultural soil or through the irrigation of farm produce with contaminated water [12]. Globally, the outbreak of farm produce related listeriosis outbreak has been reported [13]. In Texas, 10 deaths were recorded in 2010 due to listeriosis outbreak associated with chopped celery [14]. In Canada, 30 people in 2011 were infected by melons contaminated with *L*. *monocytogenes* [15]. In 2014, the outbreak of listeriosis connected to contaminated caramel apple was also recorded in California [16]. This proposes that *L*. *monocytogenes* can be spread through the water-plant-food nexus of the agricultural eco-system, and so measures to curtail the dissemination of this pathogen to the food web should be adapted including adequate pre and post-harvest activities as well as the appropriate use of antibiotics to provide protection against listeria food poisoning when the need arises.

*L*. *monocytogenes* are susceptible to most antibiotics commonly used against Gram-positive bacteria, having a cure rate of approximately 70%. However, the gravity of listeriosis can only be averted by the early administration of the right set of antibiotics [17]. Usually, combination therapy involving gentamicin and amoxicillin or ampicillin is used for the alleviation of listeriosis in humans while erythromycin, vancomycin and trimethoprim-sulfamethoxazole are drugs of choice when treating pregnant women [18,19]. A study carried out in France showed that only 1.27% human isolates out of 4816 clinical *L*. *monocytogenes* resisted the effects of antibiotics tested [20]. Notwithstanding, a growing number of antibiotic-resistant *L*. *monocytogenes* are continually documented [1,4]. Naturally occurring resistance to fosfomycin, third-generation cephalosporin and first-generation quinolones have also been documented [21]. The enormous administration of antibiotics in human and animal medicine has subjected *Listeria*. spp. to sub-therapeutic concentrations of antibiotics [22]. This causes the selective pressure that encourages the development of resistance in these pathogens especially via gene alterations or acquisition of mobile genetic elements [23]. In 1988, multidrug-resistant (MDR) *L*. *monocytogenes* was first isolated from a patient in France that suffered from meningoencephalitis [24]. In 1990, the first acquired antibiotic resistance in *L*. *monocytogenes* was described [25]. Since then, MDR strains responsible for the sporadic animal and human listeriosis recovered from food, environmental and clinical samples are been described [26–28].

Irrigation water and agricultural soil play a huge role in the incidence of antibiotic-resistant *L*. *monocytogenes* in fresh produce and the subsequent outbreak of farm produce related listeriosis [29]. This poses a threat to food safety and public health, especially with the lingering pandemic of listeriosis in South Africa and so urgent action is needed. In this paper, we report the antibiogram fingerprints of *L*. *monocytogenes* in irrigation water and agricultural soil in two DMs in the Eastern Cape Province, South Africa. To the best of our knowledge, this is the first report.

## Materials and methods

### Study area

This study was undertaken in Amathole and Chris Hani DMs in the Eastern Cape Province, South Africa. Amathole DM is situated in the central part of the Eastern Cape while Chris Hani DM is a Category C Municipality situated in the north-eastern part of the Eastern Cape. These DMs are majorly agrarian and provide a suitable investment environment for agro-processing due to their closeness to the ports of East London and Port Elizabeth. Nineteen sampling sites located within these two DMs were selected for the study and the full description of the sampling sites including their geographical coordinates are shown in S1 Table. Sample sites 1 to 14 are in Amathole DM while sample sites 15 to 20 are in Chris Hani DM. Reconnaissance visits were carried out in all the sapling sites before the commencement of the study, and permits to collect samples from some of the farms were given.

### Collection of samples, processing and enumeration of *L*. *monocytogenes*

Nineteen irrigation water samples (1000 ml) were retrieved in 1 L sterile sample bottles on a once-off basis in triplicate per sampling site and conveyed on ice to the laboratory for analysis within 6 hours of sample collection. Three series of ten-fold dilution of irrigation water samples (10^−1^, 10^−2^ and 10^−3^) was carried out as described by [30] and 100 ml of each dilution was filtered through membrane filters (MF) of 0.45 μm pores (Merck, South Africa) in triplicates, using a vacuum pump. The membrane filters were aseptically placed onto already prepared Petri-dishes containing Chromogenic Listeria Agar (ISO) Base (Oxoid Ltd, United Kingdom) supplemented with OCLA (ISO) differential supplement (Oxoid Ltd, England) and OCLA (ISO) selective supplement (Oxoid Ltd, England), conforming to the formulation described by Ottaviani and Agosti (ALOA) in ISO 11290–1:1997 [31] for the enumeration of presumptive *L*. *monocytogenes*. In the same vein, 13 agrarian soil samples (approximately 30 g) were collected in sterile plastic bags using sterile soil sampler on a once-off basis in triplicates per sampling site, kept on ice packs and conveyed to the laboratory for analysis within 6 hours. On getting to the laboratory, 1 g (dry weight) of randomly mixed soil samples were homogenised in 10 ml sterile distilled water by vortexing, after which the soil suspensions were subjected to three series of ten-fold dilution (10^−1^, 10^−2^ and 10^−3^) as described by [32]. One hundred microliter of each dilution of the soil suspensions was plated in triplicates on already prepared plates, containing Chromogenic Listeria Agar (ISO) Base (Oxoid Ltd, United Kingdom) supplemented with OCLA (ISO) differential supplement (Oxoid Ltd, England) and OCLA (ISO) selective supplement (Oxoid Ltd, England) using the spread plate method as described by [33].This conforms to the formulation described by Ottaviani and Agosti (ALOA) in ISO 11290–1:1997 for the enumeration of presumptive isolates of *L*. *monocytogenes*. All the plates were placed in the incubator, set at 37°C for 24 hours in an upturned position. After incubation, the target isolates were identified based on their morphological characteristics, counted and recorded. Blue colonies with halos were presumptive for *L*. *monocytogenes* on Chromogenic Listeria Agar (ISO) Base. The data were transformed to log (x + 1) CFU/100ml of water samples and log (x + 1) CFU/g of soil samples.

### Isolation and PCR-based confirmation of presumptive *L*. *monocytogenes*

For enrichment purposes, 25 ml of irrigation water samples and 25 g of soil samples were inoculated into 225 ml of Buffered Listeria Enrichment Broth (Oxoid, England) supplemented with Listeria Primary Selective Enrichment Supplement (Oxoid, England) after which the broth was incubated at 30°C for 48 hours. Following incubation, 1ml of the Buffered Listeria Enrichment Broth was transferred to 9ml of sterile 0.5% KOH, vortexed and spread plated on Chromogenic Listeria Agar (ISO) (Oxoid Ltd, United Kingdom) supplemented with OCLA (ISO) differential supplement (Oxoid Ltd, England) and OCLA (ISO) selective supplement (Oxoid Ltd, England). The plates were incubated at 37°C for 24 hours, after which, presumptive isolates (blue colonies with halos) were further purified on nutrient agar and overnight pure cultures were stocked in 20% glycerol at −80°C for further analysis. Upon resuscitation of glycerol stock using nutrient broth, presumptive *L*. *monocytogenes* were subjected to PCR confirmation. This was started off with DNA extraction using the boiling method as described by [34,35] with little modifications. A single colony from 24 hours grown culture on nutrient agar was transferred to 200 μl of sterile nuclease-free water. The suspension was vortexed, and the cells lysed via heating for 15 min at 100°C in an MS2 Dri-Block DB.2A (Techne, SA). The lysed cells were cooled on ice for 5 min after which the suspension was then centrifuged at 10,000 rpm for 5 min to remove cell debris using the PRISMR Centrifuge (Labnet International, Inc). Thereafter, the lysate supernatant which is the extracted DNA was used as a template for PCR assays. Molecular confirmation of presumptive *Listeria* spp. (n = 300) was done by targeting the putative phosphoribosyl pyrophosphate synthetase *(prs)* gene while the *prf*A gene was targeted for *L*. *monocytogenes* as described by [36] in a polymerase chain reaction. *L*. *monocytogenes* ATCC 9525 (ATCC; Manassas, Va., USA) was used as a positive control. The PCR mixture was made up of 12.5 μL PCR master mix (Thermo scientific (EU) Lithuana), 1.25 μL of forward and 1.25 μL of reverse primers (White Sci, SA), 2 μL of DNA template and 8 μL of PCR grade water to a final volume of 25 μL. The amplification was performed using the thermal cycler (BIORAD, Mycycler ^TM^ thermal cycler) and specific primers (S2 Table) that target *prs* and *prf*A gene. The cycling conditions were as follows: 5 min, 94°C; 33 (45 sec, 94°C; 30 sec, 56°C; 1 min, 72°C); 5 min, 72°C. The products of PCR (5 μl aliquots) were resolved in 1.5% (w/v) agarose gel (Merck, SA) that contains 0.5 μg Ethidium bromide (EtBr) (White Sci, SA) in 1X Tris-borate-EDTA (TBE) buffer (40mM Tris-HCl, 20 mM Na acetate, 1mM EDRA, pH 8.5) before being visualized under the UV Transilluminator (Alliance 4.7). A 100-bp DNA ladder (Promega, White Sci) was added to each gel during the electrophoresis as a molecular size standard which was run at 100 v for 40 minutes.

### Screening of virulence genes

The confirmed isolates were screened for 9 most commonly occurring virulence markers namely *inlA*, *inlB*, *inlC*, *inlJ*, *actA*, *hlyA*, *plcA*, *plc*B, and *iap* in a PCR based technique as described by [3] using the sets of primers shown in S3 Table. The DNA templates of confirmed isolates of *L*. *monocytogenes* were extracted using the boiling technique and the PCR reaction mixture was to a final volume of 25 μL as described above. The PCR was carried out using the protocol as follows: 5 minutes, 94°C; 35(35 seconds, 94°C; 30 seconds, 52°C; 1 minute, 72°C); 10 minutes, 72°C. The amplicons were electrophoresed and visualized as described above.

### Antibiotic susceptibility testing of isolates

The antibiotic susceptibility patterns of confirmed *L*. *monocytogenes* were analyzed using the Disk Diffusion Test as recommended by the Clinical Laboratory Standards Institute (CLSI) [37]. Eighteen antibiotics belonging to 10 classes of antibiotics frequently dispensed for the alleviation of listeriosis and other Gram-positive bacteria causing infections were used for susceptibility testing and they include; penicillin G (PG-10μg), ampicillin (AP-10μg) belonging to β-lactams, kanamycin (K-30μg), gentamycin (GM-10μg), amikacin (AK-30μg) belonging to Aminoglycosides, erythromycin (E-15μg), azithromycin (ATH-15μg) belonging to Macrolides, ciprofloxacin (CIP-5μg), norfloxacin (NOR-10μg), levofloxacin (LEV-5μg) belonging to Fluoroquinolones, nitrofurantoin (N-30μg) belonging to Nitrofurans, trimethoprim/sulfamethoxazole (TS-1.25μg/23.75μg) belonging to Sulfonamides, chloramphenicol (C-30μg) belonging to Phenicols, doxycycline (DXT-30μg), tetracycline (T-30μg) belonging to Tetracyclines, cefotaxime (CTX-30μg), cefuroxime (CXM-30μg) belonging to Cephems and linezolid (LZ-10 μg) belonging to Oxazolididones. Colonies were picked from 24 hours incubated pure culture on nutrient agar, emulsified in sterile normal saline and adjusted to match a 0.5 McFarland standard. A sterile swab was used to inoculate the mixture evenly on Mueller-Hinton agar and antibiotics transferred on the agar using a disc dispenser after which they were incubated for 24 hours at 37°C. The inhibition zones were measured to the nearest mm and the values were interpreted as “Resistant (R), Intermediate (I) or Susceptible (S)” using the standards recommended by CLSI [37] for *Staphylococci* [38].

### Evaluation of Multiple Antibiotic Resistance Phenotypes (MARPs) and Multiple Antibiotic Resistance Index (MARI) of *L*. *monocytogenes*

The multiple antibiotic-resistant phenotypes (MARPs) of *L*. *monocytogenes* were evaluated for isolates that exhibited resistance to three or more antibiotics according to the method adapted from [39]. The MDR pattern, amount of antibiotics the isolates exhibited resistance to, and the number of the observed phenotypic pattern were also described. Also, the MARI for each multidrug isolate was generated using the mathematical equation adapted from [39] which is given as:

MARindex=a/b

Where ‘a’ equals the number of antibiotics to which the isolates exhibit resistance to and ‘b’ equals the amount of antibiotics against which each isolate was tested. MARI that is equal to or greater than 0.2 shows that antibiotics are intensively used in that region and stands a high risk of promoting antibiotic resistance [40,41].

### Antibiotic resistance genes detection in *L*. *monocytogenes*

Resistance determinants were screened for in confirmed *L*. *monocytogenes* using PCR techniques. Nineteen resistance genes that code for tetracycline, sulfonamides, phenicol and aminoglycoside resistance were screened for using simplex, duplex or multiplex PCR, and these genes, their primer sequence, cycling conditions and expected amplicon size are shown in S4 Table. Also, 21 genes that code for *Amp*C β-lactamases and various variants of extended-spectrum β-lactamases (ESBLs) and carbapenemases were also screened for using simplex and multiplex PCR as described by [42] and [43] respectively. The genes, their sequences and expected amplicon sizes are shown in S5 Table. The cycling conditions used for the PCR amplification of the *Amp*C β-lactamase gene was: 4 minutes, 94°C; 30(45 seconds, 94°C; 45 seconds, 60°C; 45 seconds, 72°C);7 minutes, 72°C. For ESBL genes: 10 minutes, 94°C; 30(40 seconds, 94°C; 40 seconds, 60°C; 1 minute, 72°C);7 minutes, 72°C. The optimal annealing temperature for *bla*_GES_ and *bla*_OXA-48_ amplification was 57°C and 55°C for *bla*_VIM_, *bla*_IMP_ and *bla*_KPC_ carbapenemases genes. The PCR and electrophoresis were run as described above.

### Evaluation of the patterns of multiple antibiotic resistance genotypes (MARGs) in *L*. *monocytogenes*

The patterns of MARGs in *L*. *monocytogenes* recovered from irrigation water and agricultural soil samples harbouring multiple resistance genes ≥2 were evaluated as described by [42]. The total number of resistance genes other than β-lactamases and the total number of β-lactamases including *Amp*C and plasmid-mediated *Amp*C harboured by the isolates as well the number of observed MARGs patterns were fully described.

### Data analysis

Data obtained from this study were subjected to statistical analysis using IBM Statistical Package for Social Sciences (SPSS version 21). The comparison of the counts of *L*. *monocytogenes* in irrigation water and agricultural soil samples across the sampling sites was determined via one-way analysis of variance (ANOVA). Mean ± SD was statistically significant at *P-*values ≤0.05. Data obtained from antibiotic susceptibility tests were subjected to descriptive statistical analysis.

## Results

### The distribution of presumptive *L*. *monocytogenes* in irrigation water and soil samples

**Fig 1:**
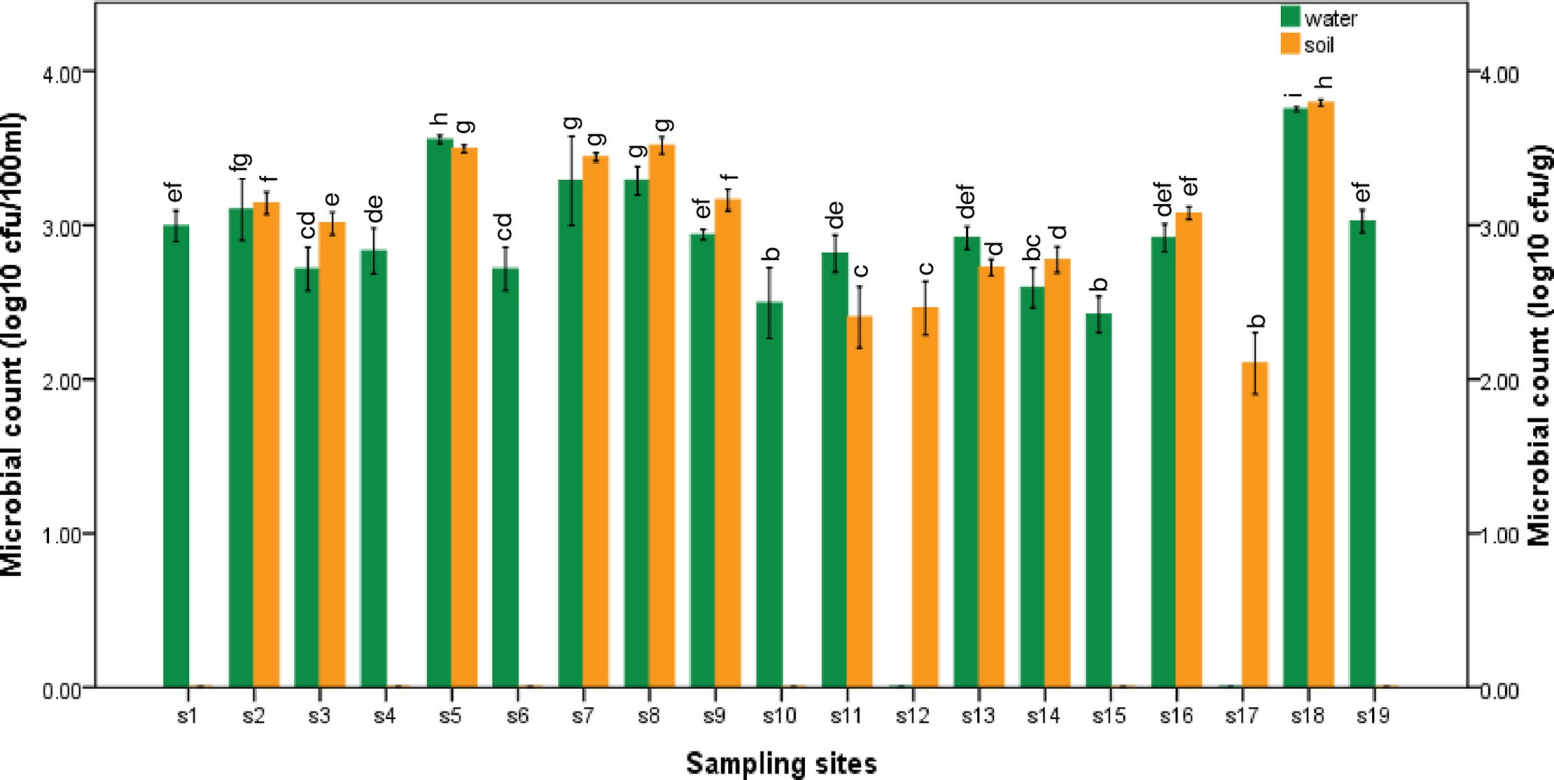
Distribution of presumptive *L*. *monocytogenes* in irrigation water (green bars) and agricultural soil (yellow bars) samples collected from the sampling sites within Amathole (s1 to s14) and Chris Hani (s15 to s19) DMs. Mean ± SD of the microbial count for the different sampling sites were statistically significant at *P* ≤ 0.05, *F* = 232.563 for irrigation water samples and *P* ≤0.05, *F* = 1196.548 for soil samples. The bars with the same colour and letter(s) are not significantly different (*P* ≥ 0.05, Duncan) across sampling sites.

The distribution of presumptive *L*. *monocytogenes* in irrigation water samples ranged from log_10_ 1.00 CFU/100ml in S12 and S17 to log_10_ 3.75 CFU/100 ml in S18 as shown in Fig 1. Also, the distribution of presumptive *L*. *monocytogenes* in soil samples collected ranged from log_10_ 2.10 CFU/g in S17 to log_10_ 3.51 CFU/g in S8 as shown in Fig 1. Soil samples were not collected from S1, S4, S6, S10, S15 and S19 due to the inaccessibility of agricultural farms using the sampled irrigation water. The distribution of *L*. *monocytogenes* in both irrigation water and soil samples collected were statistically significant (*P* ≤ 0.05).

### PCR confirmation of presumptive *L*. *monocytogenes*

Three hundred presumptive isolates of *L*. *monocytogenes* were retrieved from irrigation water and agricultural soil samples collected from the sampling sites and only 148 (49%) were positive for the *prs* gene which code for *Listeria* genus. All the positive *Listeria* spp. were further screened for the *prfA* gene which code for *L*. *monocytogenes* and only 20 (14%) were positive. Sixteen percent of irrigation water samples and 23% of agricultural soil samples were positive for *Listeria monocytogenes*.

### The prevalence of confirmed *L*. *monocytogenes* in irrigation water and agricultural soil within different sampling sites

**Fig 2:**
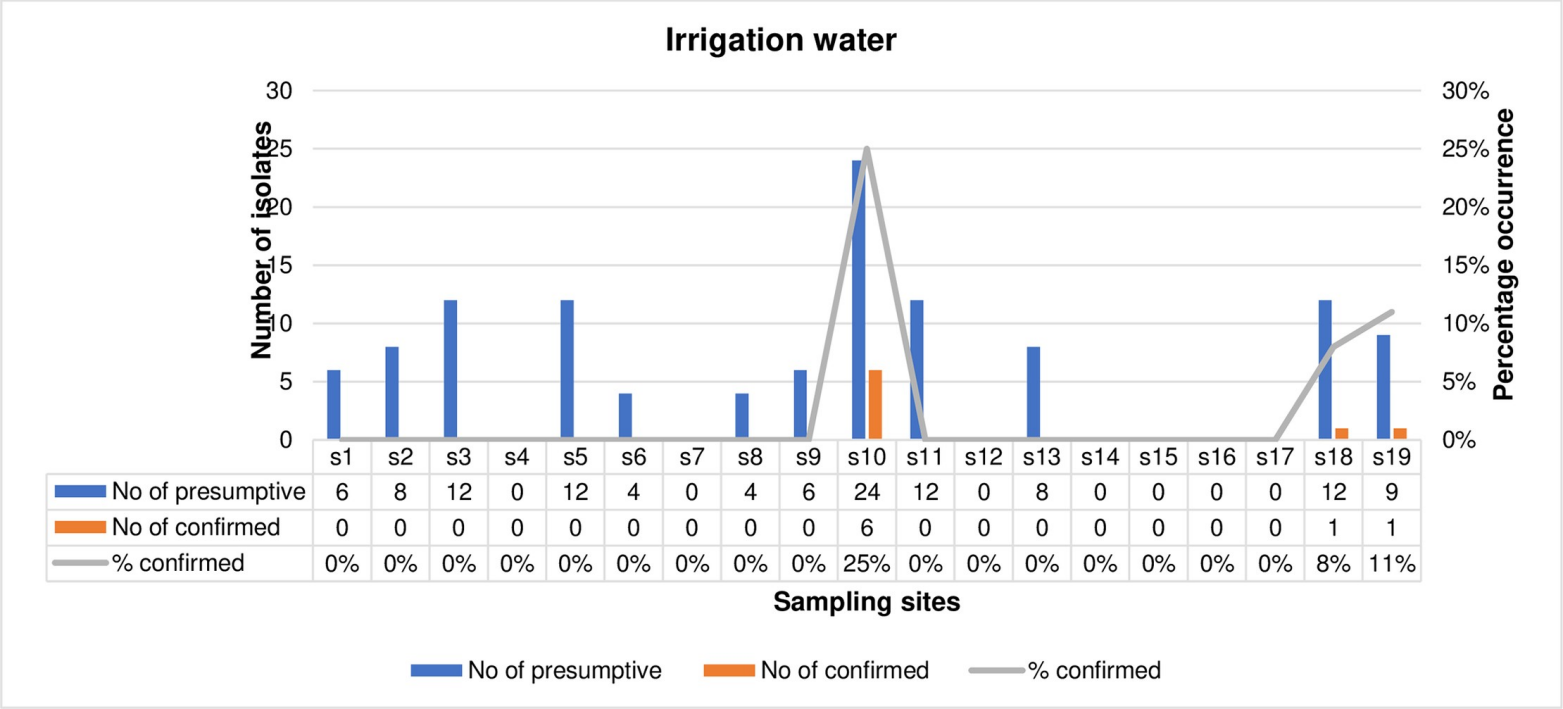
The prevalence of confirmed *L*. *monocytogenes* in irrigation water within different sampling sites.

**Fig 3:**
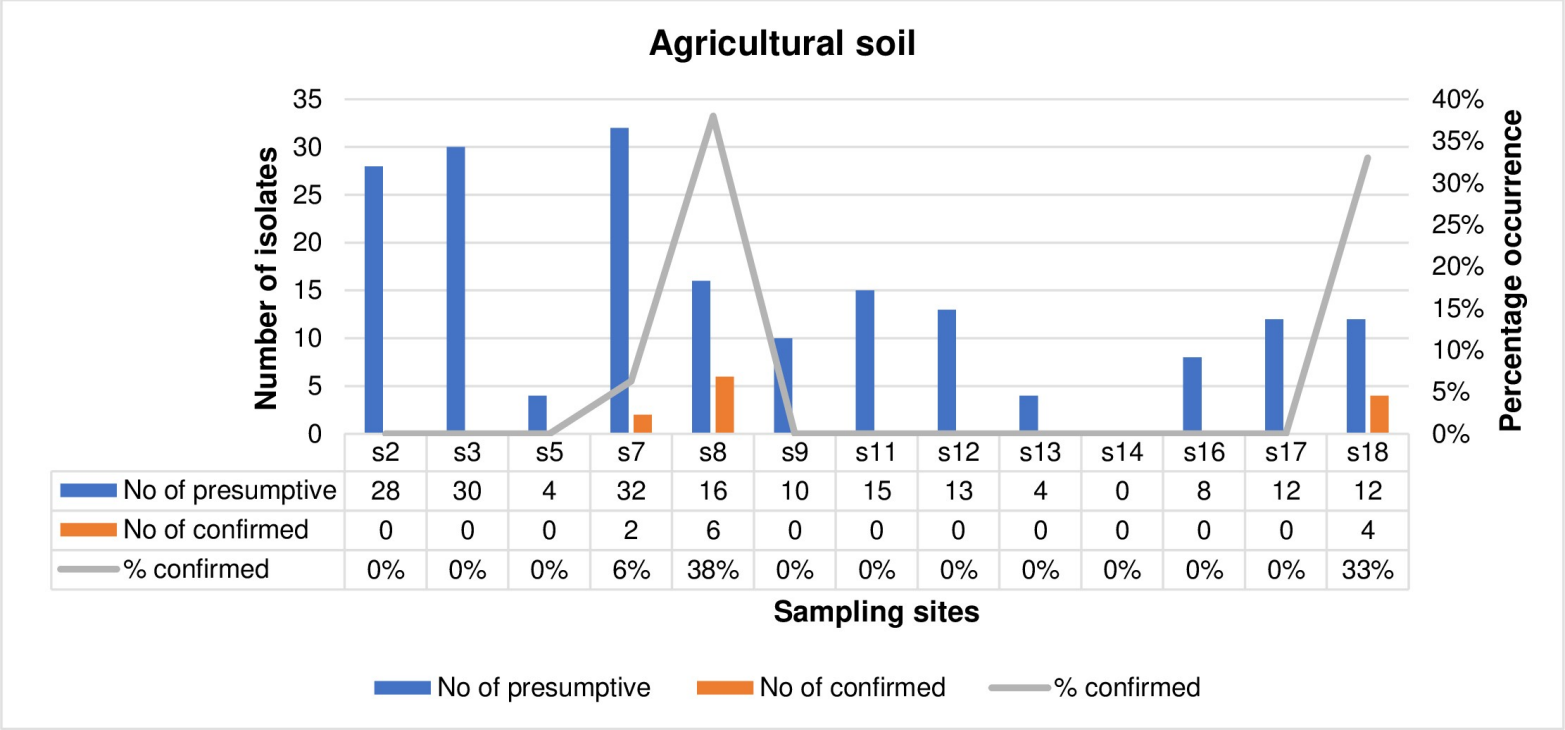
The prevalence of confirmed *L*. *monocytogenes* in agricultural soil samples within different sampling sites.

The frequency of occurrence of confirmed *L*. *monocytogenes* in irrigation water samples and agricultural soil samples is represented in Figs 2 and 3 respectively with respect to the sampling sites. The number of presumptive isolates of *L*. *monocytogenes* from irrigation water samples ranged from 0 in s4, s7, s12, s14, s15, s16 and s17 to 24 in s10 out of which 1 isolate was confirmed in s18 (8%) and s19 (11%), and 6 (25%) isolates in s10. In total, 117 presumptive isolates of *L*. *monocytogenes* were recovered from the irrigation water samples collected from all the sampling sites and only 8 (6.8%) were confirmed. Also, the number of presumptive isolates of *L*. *monocytogenes* in agricultural soil samples ranged from 0 in s14 to 32 in s7 and from this, *L*. *monocytogenes* were only confirmed in s7, s8 and s18 amounting to 2(6%), 6(38%) and 4(33%) respectively. In total, 183 presumptive isolates of *L*. *monocytogenes* were retrieved from the agricultural soil samples in all the sampling sites and only 12 (6.6%) were confirmed. In total, out of 300 presumptive isolates of *L*. *monocytogenes*, only 20 (13.5%) were confirmed and subjected to further investigations.

### Detection of virulence markers in confirmed *L*. *monocytogenes*

**Fig 4:**
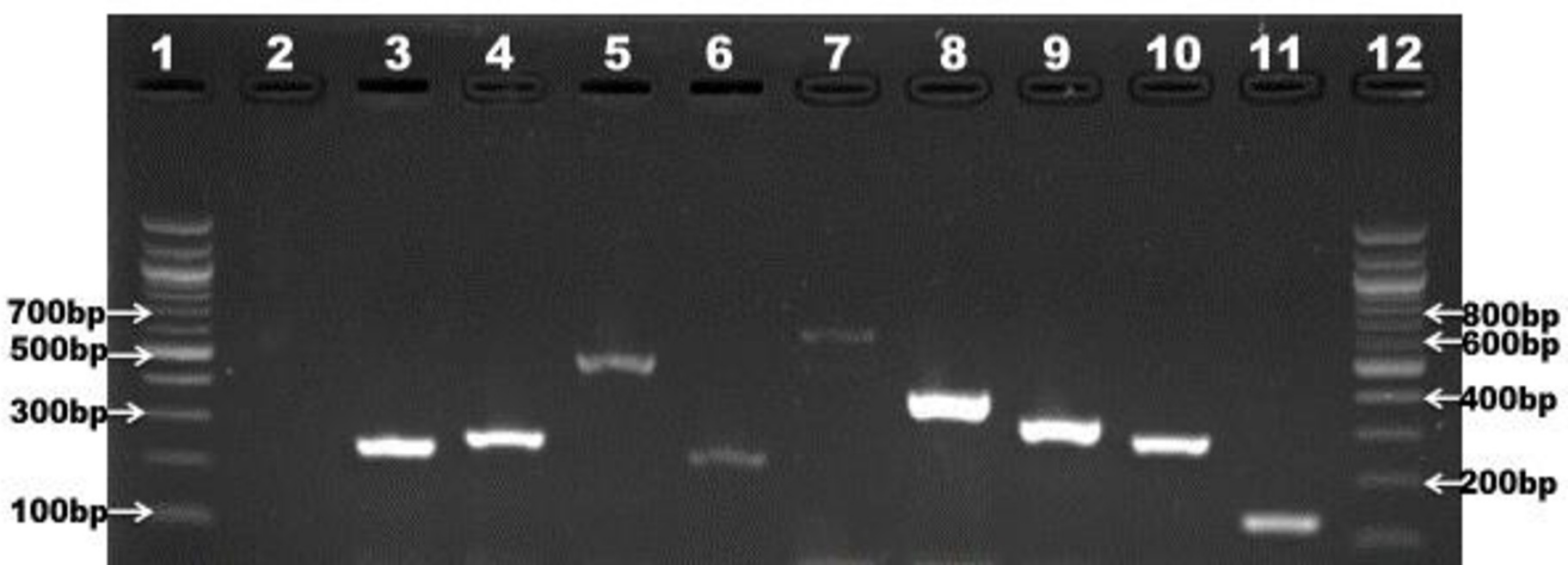
Gel picture showing multiplex PCR amplification of virulence genes; lane 3: *inlA* (256pb), lane 4: *inlB* (272bp), lane 5: *inlC* (517bp), lane 6: *inlJ* (238bp), lane 7: *actA* (650bp), lane 8: *hylA* (404bp), lane 9: *plcA* (326bp), lane 10: *plcB* (289bp), lane 11: *iap* (131bp), lane 1 and 12 represents 100bp DNA ladder and lane 2 represents negative control.

**Fig 5:**
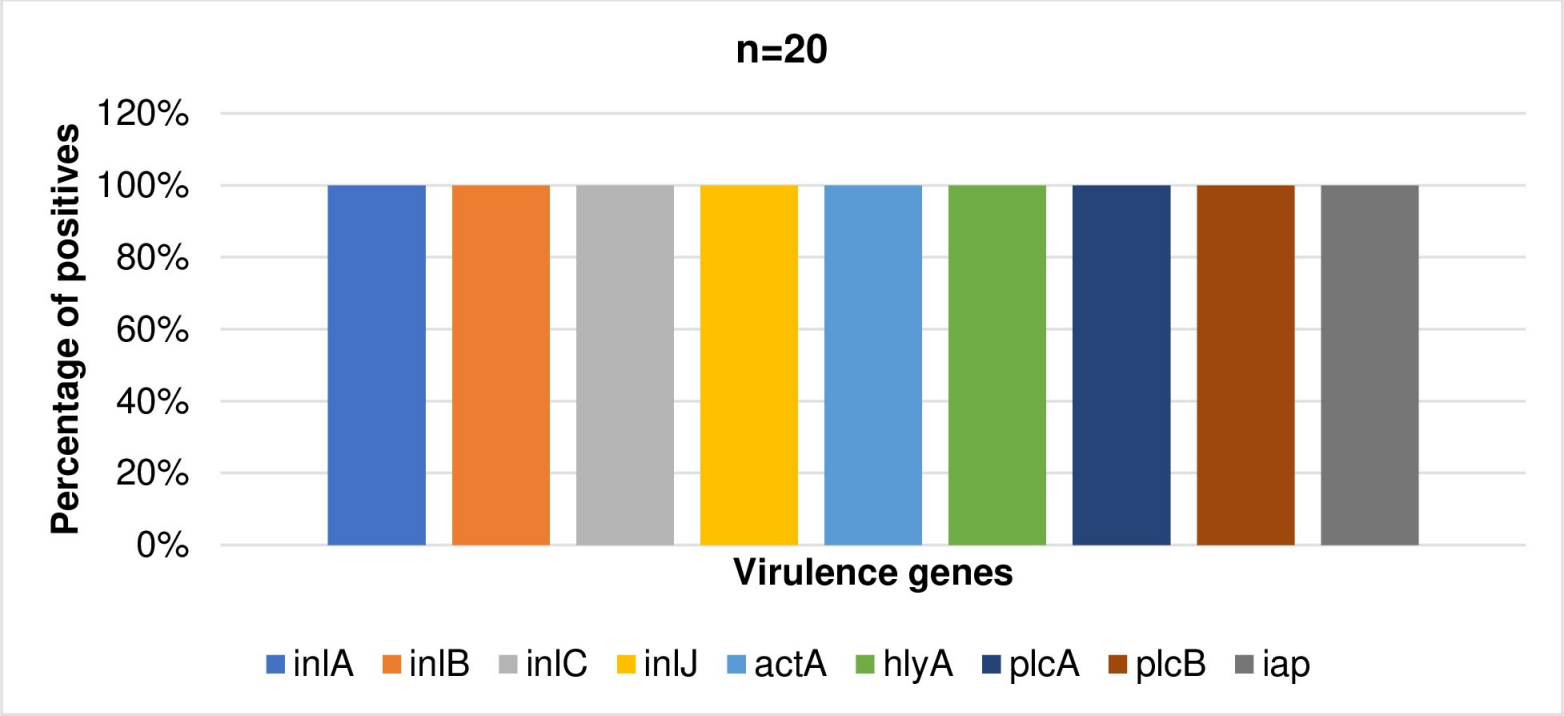
The prevalence of virulence genes in confirmed isolates of *L*. *monocytogenes* (n = 20).

Nine commonly occurring virulence markers were screened in all confirmed *L*. *monocytogenes* isolates (n = 20). All of the virulence genes were present in each isolate as shown in Fig 4. The prevalence of the virulence markers in confirmed isolates is summarized in Fig 5.

### Evaluation of antibiotic susceptibility profile of *L*. *monocytogenes* to test antibiotics

**Fig 6:**
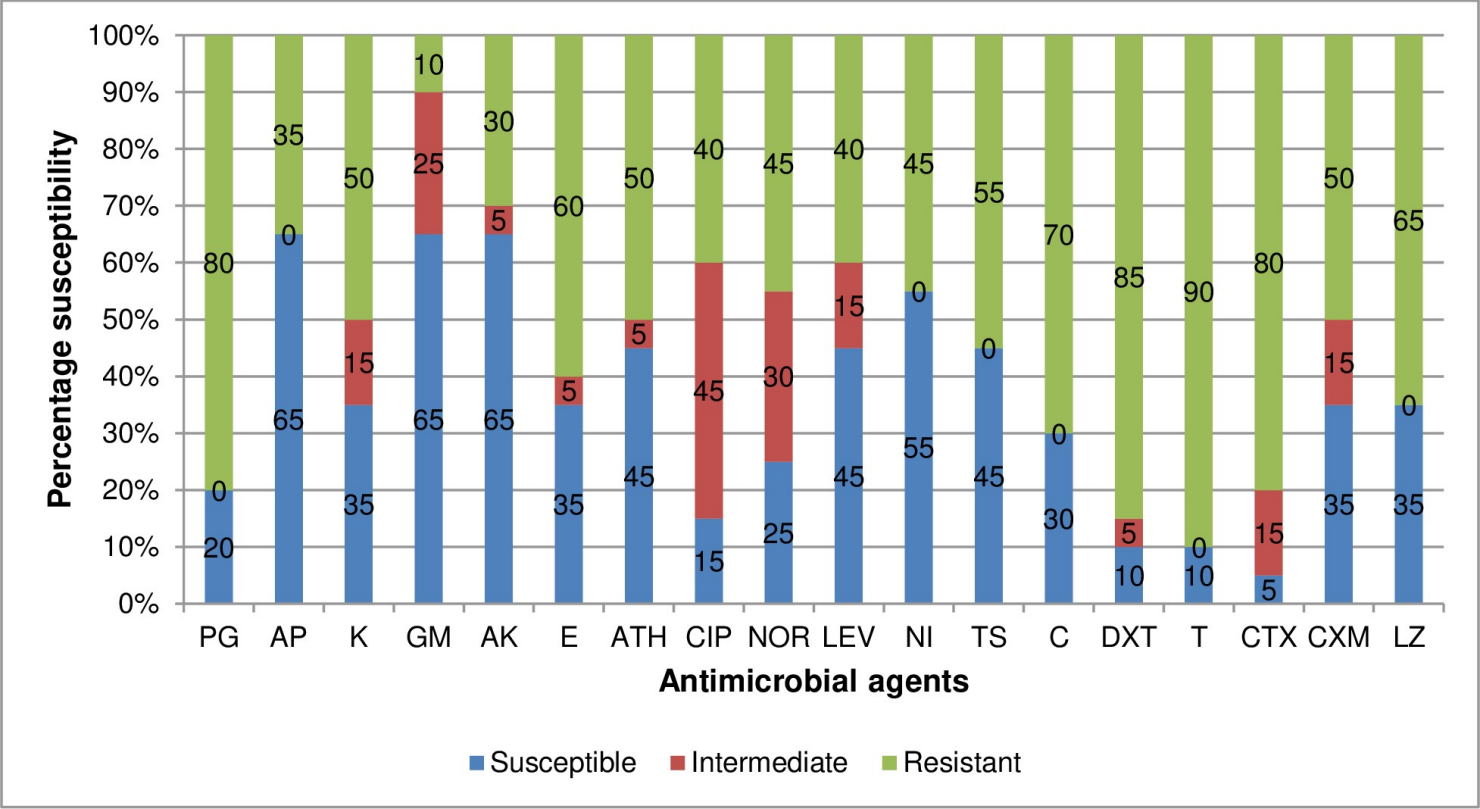
Antibiotic susceptibility profile of *L*. *monocytogenes*. PG-Penicillin G, AP- Ampicillin, K-Kanamycin, GM-Gentamycin, AK-Amikacin, E-Erythromycin, ATH- Azithromycin, CIP-Ciprofloxacin, NOR-Norfloxacin, LEV-Levofloxacin, N-Nitrofurantoin, TS-Trimethoprim/Sulfamethoxazole, C-Chloramphenicol, DXT-Doxycycline, T-Tetracycline, CTX- Cefotaxime, CXM-Cefuroxime and LZ-Linezolid.

The susceptibility profile of confirmed *L*. *monocytogenes* isolates (n = 20) against test antibiotics is shown in Fig 6. According to the results, high frequency of resistance was observed against tetracycline (90%), doxycycline (85%), cefotaxime (80%), penicillin (80%), chloramphenicol (70%), linezolid (65%), erythromycin (60%) and trimethoprim/sulfamethoxazole (55%). Varied frequency of resistance was observed against kanamycin (50%), azithromycin (50%), ciprofloxacin (40%), norfloxacin (45%), cefuroxime (50%), levofloxacin (40%). Alternatively, high susceptibility was observed against ampicillin (65%), gentamicin (65%), amikacin (65%) and nitrofurantoin (55%).

### Phenotypic antibiotic resistance pattern of each *L*. *monocytogenes* isolates

**Fig 7:**
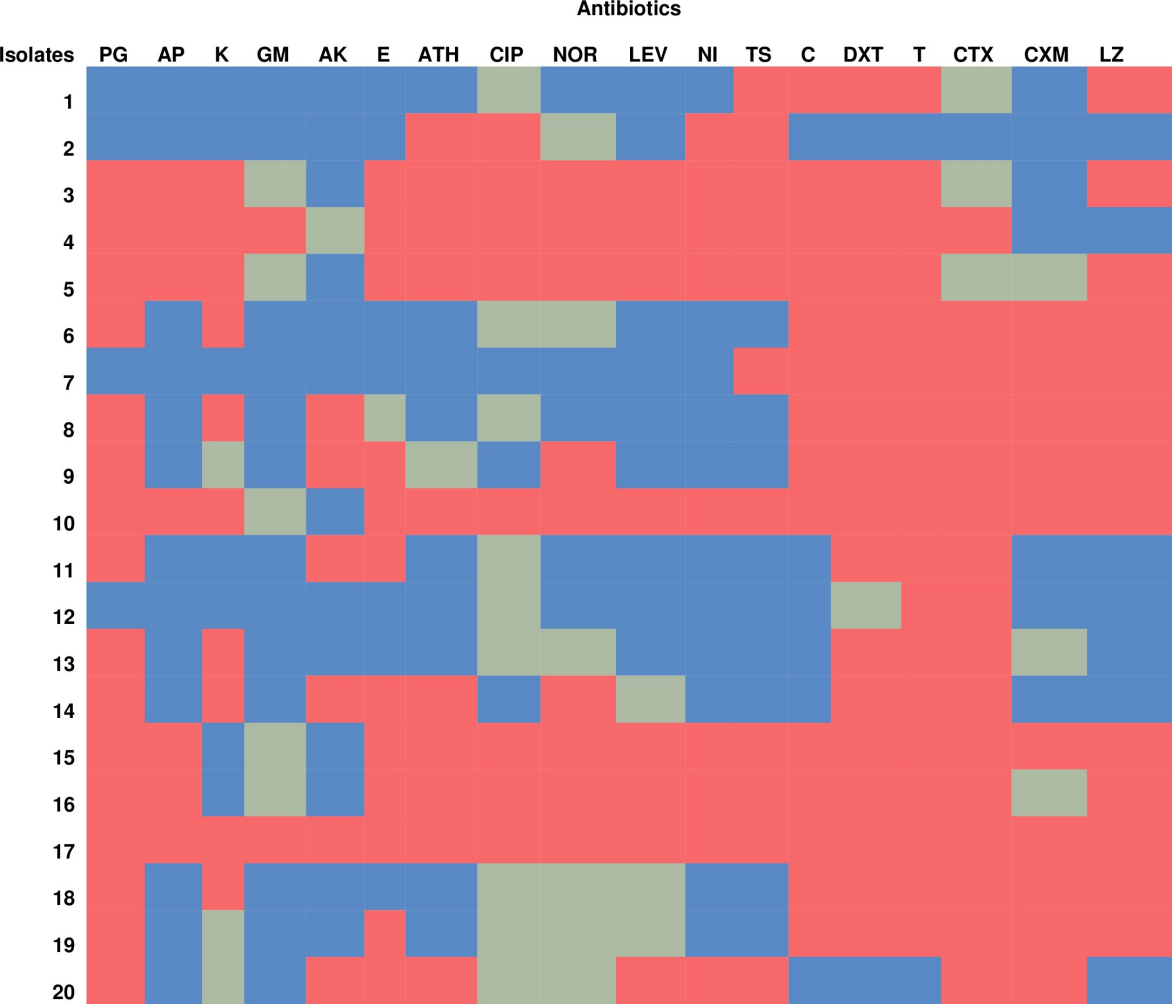
Heatmap showing the phenotypic antibiotic resistance patterns of each *L*. *monocytogenes* isolates. Colour keys indicate sensitive (Blue), Intermediate (pale green), Resistance (Red). PG-Penicillin G, AP-Ampicillin, K-Kanamycin, GM-Gentamycin, AK-Amikacin, E-Erythromycin, ATH-Azithromycin, CIP-Ciprofloxacin, NOR-Norfloxacin, LEV-Levofloxacin, N-Nitrofurantoin, TS-Trimethoprim/Sulfamethoxazole, C-Chloramphenicol, DXT-Doxycycline, T-Tetracycline, CTX-Cefotaxime, CXM-Cefuroxime and LZ-Linezolid.

The phenotypic antibiotic resistance patterns of each *L*. *monocytogenes* isolate as represented in the heatmap in Fig 7, shows the observable antibiotic resistance trait exhibited by an individual isolate of *L*. *monocytogenes*. This gives a clue on the influencing genetic traits in each isolate. Isolates 1 to 8 were recovered from irrigation water samples while isolates 9 to 20 were recovered from agricultural soil samples. According to the results, only 1% of the total isolates (isolate 17) showed resistance phenotypically to all the antibiotics tested. Isolate 3, 4 and 5 phenotypically showed almost similar resistance pattern to test antibiotics. Generally, isolates from agricultural soil samples exhibited more resistance to test antibiotics compared to isolates from the irrigation water samples. In total, about 40% of the isolates conferred resistance phenotypically to more than 50% of the antibiotics tested. The rest of the isolates conferred phenotypic resistance to at least one antibiotic.

### The patterns of MARPs and MARI of *L*. *monocytogenes* isolated from irrigation water and agricultural soil samples

The patterns of MAR phenotypes and the MAR indices of *L*. *monocytogenes* isolated from irrigation water samples and agricultural soil samples are represented in Tables 1 and 2 respectively. According to results, isolates from irrigation water exhibited 7 patterns of MARPs to antibiotics ranging from 4 to 15, most of which were uniquely observed. The MARI of the MDR isolates from irrigation water were all greater than 0.2 which is the permissible benchmark for MARI. Isolates from agricultural soil exhibited 11 patterns of MARPs to antibiotics ranging from 5 to 18, all of which were uniquely observed. The MARI of the isolates from agricultural soil were all greater than 0.2 which is the permissible benchmark for MARI.

**Table 1:** Patterns of MAR phenotypes and MAR index of *L*. *monocytogenes* isolated from irrigation water samples.

SN	MAR Phenotypes	No. of antibiotics	No. observed	MARI
1	TS-C-DXT-T-LZ	5	1	0.3
2	ATH-CIP-NI-TS	4	1	0.2
3	PG-AP-K-E-ATH-CIP-NOR-LEV-NI-TS-C-DXT-T-LZ	14	2	0.8
4	PG-AP-K-GM-E-ATH-CIP-NOR-LEV-NI-TS-C-DXT-T-CTX	15	1	0.8
5	PG-K-C-DXT-T-CTX-CXM-LZ	8	1	0.4
6	TS-C-DXT-T-CTX-CXM-LZ	7	1	0.4
7	PG-K-AK-C-DXT-T-CTX-CXM-LZ	9	1	0.5

PG-Penicillin G, AP-Ampicillin, K-Kanamycin, GM-Gentamycin, AK-Amikacin, E-Erythromycin, ATH-Azithromycin, CIP-Ciprofloxacin, NOR-Norfloxacin, LEV-Levofloxacin, N-Nitrofurantoin, TS-Trimethoprim/Sulfamethoxazole, C-Chloramphenicol, DXT-Doxycycline, T-Tetracycline, CTX-Cefotaxime, CXM-Cefuroxime and LZ-Linezolid.

**Table 2:** Patterns of MAR phenotypes and MAR index of *L*. *monocytogenes* isolated from agricultural soil samples.

SN	MAR Phenotypes	No. of antibiotics	No. observed	MARI
1	PG-AK-E-NOR-C-DXT-T-CTX-CXM-LZ	10	1	0.6
2	PG-AP-K-E-ATH-CIP-NOR-LEV-NI-TS-C-DXT-T-CTX-CXM-LZ	16	1	0.9
3	PG-AK-E-DXT-T-CTX	6	1	0.3
4	PG-K-DXT-T-CTX	5	1	0.3
5	PG-K-AK-E-ATH-NOR-DXT-T-CTX	9	1	0.5
6	PG-AP-E-ATH-CIP-NOR-LEV-NI-TS-C-DXT-T-CTX-CXM-LZ	15	1	0.8
7	PG-AP-E-ATH-CIP-NOR-LEV-NI-TS-C-DXT-T-CTX-LZ	14	1	0.7
8	PG-AP-K-GM-AK-E-ATH-CIP-NOR-LEV-NI-TS-C-DXT-T-CTX-CXM-LZ	18	1	1.0
9	PG-K-C-DXT-T-CTX-CXM-LZ	8	1	0.4
10	PG-E-C-T-CTX-DXT-CXM-LZ	8	1	0.4
11	PG-AK-E-ATH-LEV-NI-TS-DXT-CTX-CX	9	1	0.5

PG-Penicillin G, AP-Ampicillin, K-Kanamycin, GM-Gentamycin, AK-Amikacin, E-Erythromycin, ATH-Azithromycin, CIP-Ciprofloxacin, NOR-Norfloxacin, LEV-Levofloxacin, N-Nitrofurantoin, TS-Trimethoprim/Sulfamethoxazole, C-Chloramphenicol, DXT-Doxycycline, T-Tetracycline, CTX-Cefotaxime, CXM-Cefuroxime and LZ-Linezolid.

### PCR-based detection of antibiotic resistance genes

Of all the resistance determinants screened in the isolates recovered from irrigation water and agricultural soil samples, the ones detected include *tetA*, *tetB* and *tetC* tetracycline resistance encoding genes., *sulI* and *sulII* sulfonamide resistance encoding genes and *aadA* and *aac(3)-IIa* aminoglycoside resistance encoding genes. *AmpC* β-lactamase encoding genes were also detected as well as other ESBL resistance encoding genes including *bla*_TEM_, *bla*_CTX-M_ group 9 and *bla*_VEB_.

### The prevalence of antibiotic resistance genes

**Fig 8:**
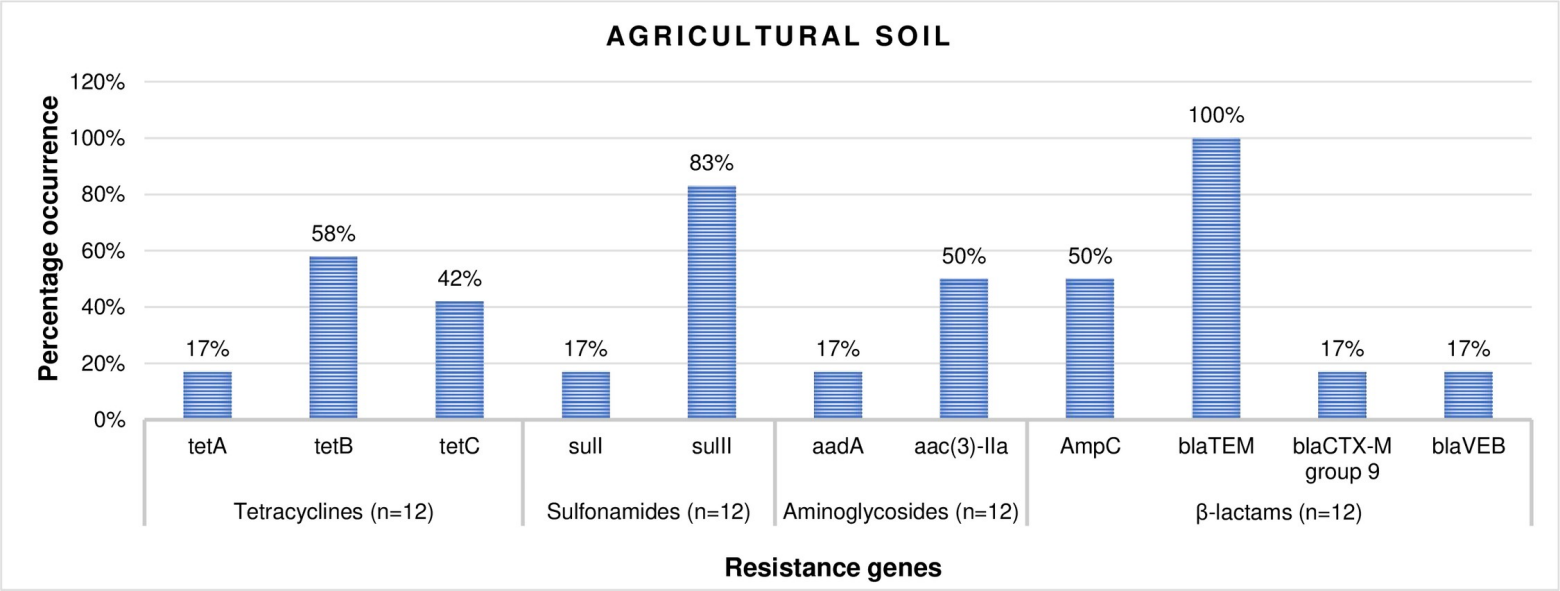
The percentage occurrence of antibiotic resistance genes in confirmed *L*. *monocytogenes* recovered from irrigation water samples. n = number of isolates screened for resistance genes.

**Fig 9:**
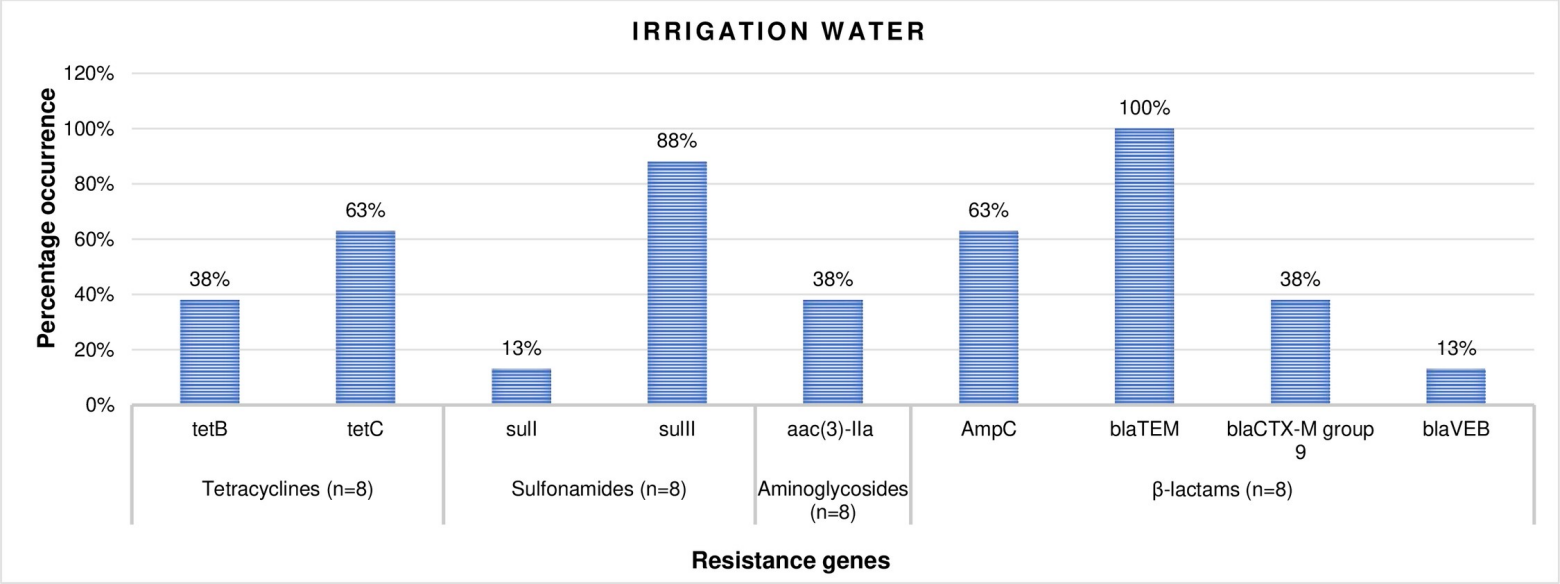
The percentage occurrence of antibiotic resistance genes in confirmed *L*. *monocytogenes* recovered from agricultural soil samples. n = number of isolates screened for resistance genes.

The percentage frequency of occurrence of the resistance genes detected in confirmed *L*. *monocytogenes* (n = 8) recovered from irrigation water samples are shown in Fig 8. According to the result, 38% and 63% of the isolates harboured *tet*B and *tet*C resistance genes respectively, 13% and 88% harboured the *sul*I and *sul*II resistance genes respectively, and 38% harboured the *aac(3)-IIa* resistance genes. Also, 63%, 100%, 38% and 13% of the isolates harboured the *AmpC*, *bla*_TEM_, *bla*_CTX-M_ group 9 and *bla*_VEB_ ESBL encoding genes respectively. In the same vein, the percentage frequency of occurrence of the resistance genes detected in confirmed *L*. *monocytogenes* (n = 12) recovered from agricultural soil samples are shown in Fig 9. According to the result, 17%, 58% and 42% of the isolates harboured *tet*A, *tet*B and *tet*C resistance genes respectively., 17% and 83% harboured *sul*I and *sul*II resistance genes respectively and 17% and 50% harboured *aadA* and *aac(3)-IIa* genes respectively. Furthermore, 50%, 100%, 17% and 17% of the isolates harboured the *AmpC*, *bla*_TEM_, *bla*_CTX-M_ group 9 and *bla*_VEB_ genes respectively.

### The patterns of multiple antibiotic resistance genotypes (MARGs) in *L*. *monocytogenes*

The MARGs patterns in *L*. *monocytogenes* recovered from irrigation water and agricultural soil samples are shown in Tables 3 and 4 respectively. According to the results, isolates from irrigation water samples exhibited 5 patterns of MARGs to resistance genes ranging from 3 to 5 and made up of both β-lactamases and non β-lactamases. Most of the MARGs patterns were uniquely observed. Isolates from agricultural soil samples exhibited 6 patterns of MARGs to resistance genes ranging from 3 to 9 and made up of both β-lactamases and non β-lactamases. Most of the MARGs patterns were observed in double and only one pattern was observed uniquely and in triplicate.

**Table 3:** Patterns of MAR genotypes of *L*. *monocytogenes* isolated from irrigation water samples.

SN	MAR Genotypes	No. of resistance genes other than β-lactamases	No. of β-lactamases including *Amp*C and p*Amp*C	No. observed
1	*tet*C*- sulII- Amp*C*- bla*_TEM_*- bla*_CTX-M_^*a*^	2	3	2
2	*tet*C*- sulII- Amp*C*- bla*_TEM_*- bla*_VEB_	2	3	1
3	*tet*C*- sulII- Amp*C*- bla*_TEM_	2	2	1
4	*tet*C*- sulII- bla*_TEM_*- bla*_CTX-M_^*a*^	2	2	1
5	*tet*B*- sulII- aac(3)-IIa- bla*_TEM_	2	1	1

Key

a = group9

**Table 4:** Patterns of MAR genotypes of *L*. *monocytogenes* isolated from agricultural soil samples.

SN	MAR Genotypes	No. of resistance genes other than β-lactamases	No. of β-lactamases including *Amp*C and p*Amp*C	No. observed
1	*tet*B*- aac(3)-IIa- Amp*C*- bla*_TEM_	2	2	2
2	*tet*B*- sulII- aac(3)-IIa- Amp*C*-bla*_TEM_	2	3	2
3	*tet*B*- sulII- aac(3)-IIa- bla*_TEM_	2	2	2
4	*tet*A*- tet*C*- sulI- sulII- aadA- Amp*C- *bla*_TEM_- *bla*_CTX-M_^*a*^ - *bla*_VEB_	5	4	2
5	*tet*C*- sulII- bla*_TEM_	2	1	3
6	*tet*B*- sulII- bla*_TEM_	2	1	1

Key

a = group9

## Discussion

In this study, the distribution of presumptive *L*. *monocytogenes* in irrigation water and agricultural soil samples corroborates with the findings of [44,45]. Currently, there is no standard guideline present for the counts of *L*. *monocytogenes* in irrigation water and agricultural soil in South Africa, thus the standard for faecal coliforms (0 CFU/100 ml) set for domestic water [46], was used in this study. Based on this benchmark, the quality of irrigation water across the sampling sites asides the irrigation water samples collected from s12 and s17 fell short of the acceptable limits, thus disqualifying their use for agricultural practices. Irrigation water in s12 and s17 are sourced from treated municipal water and groundwater respectively. Due to their enclosed nature, water from these sources are less prone to contamination, therefore, it is highly recommended to source irrigation water from these sources. Unfortunately, the agricultural soil is open to several contamination sources including manure, runoffs, contaminated irrigation water and so on, thus making it a receptacle of numerous pathogens including *L*. *monocytogenes*.

Studies on the prevalence of *L*. *monocytogenes* in irrigation water and agricultural soil is rare. However, the prevalence of *L*. *monocytogenes* obtained in this study (14%) is relatively low when related to the findings of [44,47] in surface water bodies and WWTPs effluents, but also higher than 2.3% in Chinese foods in the studies of [3]. In irrigation water samples, the highest prevalence was in s10 at a prevalence rate of 25%. This was followed by s19 at a prevalence rate of 11% and then, s18 at a prevalence rate of 8%. The anthropogenic activities within these sampling sites which include animal intrusion and dumping of refuse in sources of the irrigation water may have influenced the increased occurrence of *L*. *monocytogenes* within these sites, thus increasing the chances of contaminating the fresh produce that will be irrigated by the water. In agricultural soil samples, a high prevalence rate of 38% was noticed in s8 followed by the prevalence rate of 33% noticed in s18. In s8, soil amendment was done using improperly composted cow dung and this may have influenced the high occurrence rate of *L*. *monocytogenes* in the sample site. In s18, dairy cattle were grazed freely on the soil were their fodder is grown. They feed and defecate at the same time, shedding ample amounts *L*. *monocytogenes* to the soil, corroborating to the reports of [48–50]. This increases the risk for contaminating fodders, agricultural milieus and raw food especially at the primary stages of food production, thus posing serious problems to one-health as it impacts animal, human and environmental health negatively.

In this study, 9 virulence factors which impact the pathogenicity of *L*. *monocytogenes* were investigated. We discovered that *inlA*, *inlB*, *inlC*, and *inlJ* internalin genes which stimulates the adhesion and internalization of *L*. *monocytogenes* within the host's epithelial cells [51] occurred in 100% of the isolates recovered from both irrigation water and agricultural soil samples. This is in tune with the findings of [3], except for *inlB* where they observed a lower prevalence of 71.4%. The high prevalence of the internalin virulence genes in this study corroborates with the findings of [52–54]. However, a varying prevalence of *inlB* ranging from 45% to 100% was noticed in other studies and this shows that the prevalence of *inlB* is not consistent in all *L*. *monocytogenes*. A 100% prevalence of the *actA* gene, which stimulates actin polymerization and causes *L*. *monocytogenes* to spread in the host cells was observed in this study. This rate is quite higher than that observed in the study of [3] which is 95% and much more higher than that obtained in the studies of [55]. The prevalence of the *hylA* and *plcB* genes in the present study was 100%. The *hylA* and *plcB* mediate the release of the bacterial cells into the cytoplasm of the host and promotes the escape of the bacteria from bound vacuoles and the surface protein actin A respectively [8]. This rate is in tune with the prevalence rate observed in the study of [3]. In this study, *plcA* and *iap* which code for phospholipase and invasion-associated protein respectively, occurred in 100% of the isolates. This is also higher than the prevalence noticed in other studies [53,55–57]. The variations in the prevalence of virulence markers detected in this study and that observed in other studies may be due to the differences in the origin or source of the isolates. Environmental isolates have the tendencies to be more virulent because they are constantly exposed to single or multiple sub-lethal stress factors such as osmotic stress, cold stress, high hydrostatic pressure, acid stress, and desiccation stress which over time enhances their survival and pathogenesis [58]. This shows that *L*. *monocytogenes* isolated in this study are very virulent and capable of causing foodborne disease outbreak when introduced into the food web.

It has been reported that *L*. *monocytogenes* are generally susceptible to an extensive array of antibiotics [59]. However, recent studies have shown a tremendous reduction in their susceptibility to several antibiotics in many regions [60,61]. In this study, *L*. *monocytogenes* showed higher frequencies of resistance to tetracycline, doxycycline, cefotaxime, penicillin, chloramphenicol, linezolid, erythromycin and trimethoprim/sulfamethoxazole. This suggests that these drugs should not be dispensed to listeriosis patients. On the other hand, a high frequency of isolates was susceptible to ampicillin, gentamicin, amikacin and nitrofurantoin.These antibiotics may be efficaciously administered to suspected cases of listeriosis. No wonder [18] and [19] suggest that a combination therapy involving gentamicin and amoxicillin or ampicillin should be used for the remediation of human listeriosis. In this study, we observed 65% susceptibility to ampicillin and 50% susceptibility to cefuroxime which are drugs of choice used in the alleviation of listeriosis. Our findings were quite contrary to the verdicts of [62–64] as they noticed a much higher frequency of resistance to these group of antibiotics. These variations suggest that the indiscriminate usage of these antibiotics which most times encourages the development of resistance varies with different regions and sample types. From the results of phenotypic antibiotic resistance pattern obtained from this study, it is shown that isolates from agricultural soil exhibited more resistance to isolates from irrigation water. This shows that most of the manure that was used to improve the fertility of the soil contained partially broken antibiotics which may have caused the selective pressure that led to the increased rate of antibiotic resistance to test antibiotics. We noticed 7 patterns of MARPs among MDR isolates of *L*. *monocytogenes* recovered from irrigation water, showing resistance to antibiotics ranging from 5 to 15. They all occurred uniquely except for 1 pattern that occurred twice. In isolates recovered from agricultural soil, 11 patterns of MARPs which occurred uniquely among MDR isolates of *L*. *monocytogenes* to antibiotics ranging from 5 to 18 were observed. This is similar to the findings of [44,65]. However, [64] observed more of single resistance compared to multiple resistance. MDR in *L*. *monocytogenes* is tied to the misuse of antibiotics, which in extension causes the selective pressure which induces the emergence of resistance and the dissemination of resistance determinants among *L*. *monocytogenes* and close species such as *Staphylococcus*, *Enterococcus* and *Streptococcus* species [66]. The MARI of all MDR *L*. *monocytogenes* were all greater than 0.2. this implies that the origin of the isolates are from high-risk environments where antibiotic resistance selective pressure is high.

This study reveals a high occurrence rate of resistance determinants including *AmpC* and ESBLs in isolates recovered from irrigation water and agricultural soil. The highest prevalence was noticed for *bla*_TEM_ ESBL which occurred in 100% of the isolates recovered from irrigation water and agricultural soil samples. Although there is a dearth of information with regards to the occurrence of ESBLs in *L*. *monocytogenes* recovered from irrigation water and agricultural soil samples, a like prevalence was noticed for *E*. *coli* in the aquatic environment [42,67]. Surprisingly, the prevalence of other ESBLs including *bla*_CTX-M_ group 9 and *bla*_VEB_, as well as the *Amp*C genes was relatively high. Recently, beta-lactam antibiotics are the most extensively used antibiotics because of their low toxicity and effectiveness in the alleviation of several infectious diseases [68]. This must-have caused the increased occurrence rate of ESBLs observed in the study., thus posing a grave risk to global health. High prevalence of other resistance factors including *tet*A, *tet*B, *tet*C, *sul*I, *sul*II, *aad*A and *aac(3)-IIa* resistance genes was also noticed in the present study, aside from *tet*A and *aad*A which only occurred in isolates recovered from agricultural soil samples. Some researchers have proofed that certain agricultural activities such as the application of animal dung on farm soils expand the rate of intrinsic resistance in soil [69,70]. The presence of ARGs on the surface of vegetables and fruits have also been reported [71–74], which is related to the fact that the group of bacteria found on plant surfaces can be influenced by various antibiotic-resistant strains found in the soil or in the water used for the irrigation of the plants. To make matters worse, *L*. *monocytogenes* isolated from irrigation water and agricultural soil samples harboured multiple (2 and above) antibiotic resistance genes which are made up of ESBLs including *AmpC* and non-ESBLs. The most prominent pattern of the MARGs was “*tet*C*- sulII- Amp*C*- bla*_TEM_*- bla*_CTX-M_ group 9^*”*^ which occurred twice in the isolates recovered from irrigation water samples and “*tet*C*- sulII- bla*_TEM_” which occurred three times in isolates recovered from agricultural soil samples. A combination of ESBLs and non ESBLs in MDR *L*. *monocytogenes* can result in grave consequences including economic and clinical backlog to health care systems, government, patients, and their families.

## Conclusion

The findings of this study demonstrate that MDR *L*. *monocytogenes* occur in irrigation water and agricultural soil samples retrieved from Amathole and Chris Hani DMs, and may be disseminated to fresh produce causing food safety issues. The prevalence of *L*. *monocytogenes* recovered from irrigation water and agricultural soil samples in this study was quite low when compared to other studies. All the isolates harboured the 9 key virulence markers responsible for their pathogenicity. This shows that the isolates are very lethal coupled with the fact that they exhibited multidrug resistance, harbouring multiple resistance genes including the ESBLs and capable of causing an outbreak. We recommend that antibiotics should be used prudently in human and veterinary medicine as well as in animal husbandry. We also recommend that manure used for soil amendment be properly treated before applied to the soil. The effluents of WWTPs should be properly treated before being discharged into surface water bodies where irrigation water may be sourced from. Epidemiological studies to source track and elucidate the relatedness of the isolates in irrigation water, agricultural soil, fresh produce and in humans should be carried out.

## Supporting information

S1 Table Description of sampling points. (PDF)

S2 Table Primer sequence and expected amplicon size used for the detection of *Listeria* spp and *L. monocytogenes*. (PDF)

S3 Table The primer sequence and expected amplicon size used for the screening of virulence genes in *L. monocytogenes*. (PDF)

S4 Table The primer sequence and expected amplicon size used for the screening of resistance genes in *L. monocytogenes*. (PDF)

S5 Table The primer sequence and expected amplicon size used for the screening of *AmpC* β-lactamase [65] and ESBLs in *L. monocytogenes* [43]. (PDF)

S1 Fig Gel picture showing the molecular amplification of *prs* (370 bp) gene of *Listeria* spp isolated from irrigation water and soil samples collected. Lane 1 represents 100bp DNA ladder, lane 2 represents positive control (*L*. *monocytogenes* ATCC 9525*)*, lane 3 represents negative control and lane 4 to lane 13 represents some of the positive isolates.
(PDF)

S2 Fig Gel picture showing the molecular amplification of *prfA* (274 bp) gene of L. monocytogenes. Lane 1 represents 100bp DNA ladder, lane 2 represents positive control (*L*. *monocytogenes* ATCC 9525*)*, lane 3 represents negative control and lane 4 to lane 14 represents some of the positive isolates.
(PDF)

S1 Raw images Raw gel pictures showing the confirmation of *Listeria* genus, *Listeria monocytogenes* and virulence genes. (PDF)
