# Supplementary material for: Characterization of antibiogram fingerprints in Listeria monocytogenes recovered from irrigation water and agricultural soil samples
Source: PLoS One. 2020 Feb 10;15(2):e0228956. doi: 10.1371/journal.pone.0228956 (PMC7010277; doi:10.1371/journal.pone.0228956)
Supplement: S1 Table — (PDF) [file pone.0228956.s001.pdf]

S1 Table: Description of sampling points.

District Municipality	Sample sites	Coordinates	Samples collected	Description of samples	Anthropogenic activities
Amathole	S1	S32°46.922[1] E026°50.799[1]	Irrigation water	Irrigation water is sourced from a river. Soil samples were not collected due to the inaccessibility of agricultural farms	Animal intrusion, dumping of refuse, recreational activities, swimming, and domestic activities in the source river.
	S2	S32°46.857[1] E026°51.373[1]	Irrigation water and soil	Irrigation water is sourced from a dam. Type of irrigation used is a sprinkler irrigation system. Irrigation is done once in 3 days between 8 am and 3 pm on each day. Soil type is loam and crops grown on it include cabbage, broccoli, butternut, spinach and lettuce.	Animal intrusion in the dam. Soil amendment is with inorganic fertilizer (LAN).
	S3	S32°43.48[1] E027°1.32[1]	Irrigation water and soil	Irrigation water is sourced from an artificial pond which receives water from both rainfall and downstream river. Type of irrigation used is the sprinkler irrigation system. Irrigation is done once a day between 8 am and 12 pm, and none on rainy days. Soil type is clay-loam and crops grown on it include cabbage, broccoli, lettuce and onions.	Animal intrusion in the pond and source river. Soil amendment is with livestock waste.
	S4	S32°45.2[1] E27°19.07[1]	Irrigation water	Irrigation water is sourced from a dam. Soil samples were not collected due to the inaccessibility of agricultural farms	Animal intrusion and domestic use of water.

S5	S32°48.45[l]<br><br>E26°59.27[l]	Irrigation water and soil	Irrigation water is sourced from an artificial pond, which primarily receives water from the effluents of a poultry and piggery farm. Type of irrigation used is the sprinkler irrigation system. Irrigation is done once every day between 8 am and 5 pm except on rainy days. Soil type is loam and crops grown on it include spinach and cabbage.	Discharge of swine and poultry effluents into the pond. Soil amendment is with poultry and swine faeces.
S6	S32°35.412[l]<br><br>E026°57.793[l]	Irrigation water	Irrigation water is sourced from a river. Soil samples were not collected due to the inaccessibility of agricultural farms	Animal intrusion, recreational activities, swimming, and domestic activities in the source river.
S7	S32°38.157[l]<br><br>E026°55.954[l]	Irrigation water and soil	Irrigation water is sourced from a river. Type of irrigation used is a sprinkler irrigation system. Irrigation is done twice a week between 8 am and 4 pm each day. Soil type is clay and crops grown on it include pumpkin.	Animal intrusion, washing of cloths, bathing and other domestic activities in the source river. Soil amendment is with organic compost of livestock origin.
S8	S32°39.202[l]<br><br>E026°55.301[l]	Irrigation water and soil	Irrigation water is sourced from a river. Type of irrigation used is a sprinkler irrigation system. Irrigation is done twice a day between 8 am and 12 pm and between 2 pm and 5 pm and none on days with heavy rainfall. Soil type is clay-loam and crops grown on it include cabbage, lettuce and spinach.	Animal intrusion in both river and close to farm sites. Soil amendment is with cow dung.

S9	S32°40.998^{l} E026°54.062^{l}	Irrigation water	Irrigation water is sourced from a dam. Type of irrigation used is a sprinkler irrigation system. Irrigation is done once a day and none on days with heavy rainfall.	Animal intrusion
	S32°43.259^{l} E026°52.707^{l}	Soil	Soil type is loamy. Crops grown on it include lettuce and spinach.	Soil amendment is with composted animal waste.
S10	S22°45.617^{l} E026°51.450^{l}	Irrigation water	Irrigation water is sourced from a river. Soil samples were not collected due to the inaccessibility of agricultural farms	Animal intrusion, washing of cloths, and fishing.
S11	S32°45.618^{l} E026°51.428^{l}	Irrigation water and soil	Irrigation water is sourced from a river and manually irrigated on a backyard farm using watering cans. Soil type is loam and crops grown on it include spinach.	Animal intrusion, swimming, and washing of clothes in the source river. No soil amendment
S12	S32°46.616^{l} E026°50.203^{l}	Irrigation water and soil	Irrigation water is sourced from treated municipal water. Type of irrigation used is a sprinkler irrigation system. Irrigation is done once a day from 8 am to 1 pm. Soil type is clay-loam and crops grown on it include cabbage, lettuce, onions and spinach.	No visible anthropogenic activity in the water source. Soil amendment is done using inorganic fertilizer (LAN).

	S13	S32°35.783′<br>E027°26.880′	Irrigation water and soil	Irrigation water is sourced from a river. Type of irrigation used is the sprinkler irrigation system. Irrigation is done once a day from 8 am to 12 pm. Soil type is clay-loam and crops grown on it include cabbage, broccoli, lettuce and spinach.	Animal intrusion in the river. Soil amendment is with inorganic fertilizer (LAN).
	S14	S32°37.165′<br>E027°26.418′	Irrigation water and soil	Irrigation water is sourced from a dam. Type of irrigation used is a sprinkler irrigation system. Irrigation is done once a day. Soil type is clay and crops grown on it include cabbage, lettuce and spinach.	No observed anthropogenic activity in the dam. Soil amendment is with inorganic fertilizer (LAN).
Chris Hanni	S15	S32°19.375′<br>E027°08.543′	Irrigation water	Irrigation water is sourced from a river. Soil samples were not collected due to the inaccessibility of agricultural farms	Animal intrusion
	S16	S32°01.927′<br>E027°04.619′	Irrigation water and soil	Irrigation water is sourced from a river. Type of irrigation used is the sprinkler irrigation system. Irrigation is done once a day. Soil type is loam and crops grown on it include lettuce and spinach.	Animal intrusion. Soil amendment is with organic compost of animal origin
	S17	S31°55.297′<br>E026°49.882′	Irrigation water and soil	Irrigation water is sourced from borehole groundwater. Types of irrigation used include sprinkler and drip irrigation system. Irrigation is done twice a day. Soil type is clay-loam and crops grown on it include cabbage, lettuce, broccoli and spinach.	No anthropogenic activity in the water source. Soil amendment is with inorganic fertilizer (LAN).

S18	S31°53.203[l]<br>E026°47.764[l]	Irrigation water and soil	Irrigation water is sourced from a river. Type of irrigation used is the centre pivot irrigation system. Irrigation is done once a day. Soil type is loam and crops grown on it is fodder	Animal intrusion, receiving shed of WWTP effluent. Soil amendment is with inorganic fertilizer (LAN)
S19	S32°00.855[l]<br>E027°35.597[l]	Irrigation water	Irrigation water is sourced from a river. Soil samples were not collected due to the inaccessibility of agricultural farms	Animal intrusion, dumping of refuse.

Note: For confidentiality sake, sampling sites are coded with S1-S19. All the information recorded in this table were gotten from the onsite survey as well as from farm owners and workers. Coordinates for each sampling site were retrieved using the “etrex-LEGENDH” GPS equipment. WWTP: Wastewater treatment plant. LAN: limestone ammonium nitrogen.
