# Supplementary material for: Characterization of antibiogram fingerprints in Listeria monocytogenes recovered from irrigation water and agricultural soil samples
Source: PLoS One. 2020 Feb 10;15(2):e0228956. doi: 10.1371/journal.pone.0228956 (PMC7010277; doi:10.1371/journal.pone.0228956)
Supplement: S2 Table — (PDF) [file pone.0228956.s002.pdf]

S2 Table: Primer sequence and expected amplicon size used for the detection of *Listeria* spp and *L. monocytogenes.*

Target genes	Primer sequence (5′-3′)	Amplicon size (bp)	Reference
prs	F: GCTGAAGAGATTGCGAAAGAAG<br>R: CAAAGAAACCTTGGATTTGCGG	370	(Jami et al., 2010)
*prf*A	F: GAT ACA GAA ACA TCG GTT GGC<br>R: GTGTAA TCT TGA TGC CAT CAG	274	(Jami et al., 2010)
