# Supplementary material for: Characterization of antibiogram fingerprints in Listeria monocytogenes recovered from irrigation water and agricultural soil samples
Source: PLoS One. 2020 Feb 10;15(2):e0228956. doi: 10.1371/journal.pone.0228956 (PMC7010277; doi:10.1371/journal.pone.0228956)
Supplement: S3 Table — (PDF) [file pone.0228956.s003.pdf]

S3 Table: The primer sequence and expected amplicon size used for the screening of virulence genes in *L. monocytogenes*

Virulence Genes	Primer sequence (5′-3′)	Amplicon size (bp)	Reference
inlA	inlAF: CCTAGCAGGTCTAACCGCAC<br>inlAR: TCGCTAATTTGGTTATGCCC	256	(Coroneo et al., 2016)
inlB	inlBF: TGATGTTGATGGAACGGTAAT<br>inlBR: CTCGTGGAAGTTTGTAGATGC	272	(Du et al., 2017)
inlC	inlCF: AATTCCCACAGGACACAACC<br>inlCR: CGGGAATGCAATTTTTCACTA	517	(Liu et al., 2007)
inlJ	inlJF: TGTAACCCCGCTTACACAGTT<br>inlJR: AGCGGCTTGGCAGTCTAATA	238	(Liu et al., 2007)
actA	actAF: CCAAGCGAGGTAAATACGGGA<br>actAR: GTCCGAAGCATTTACCTCTTC	650	(Lomonaco et al., 2012)
hlyA	hlyF: ATCATCGACGGCAACCTCGGAGAC<br>hlyR: CACCATTCCCAAGCTAAACCAGTGC	404	(Du et al., 2017)
plcA	plcAF: CTCGGACCATTGTAGTCATCTT<br>plcAR: CACTTTCAGGCGTATTAGAAACGA	326	(Lomonaco et al., 2012)
plcB	plcBF: AATATTTCAATCAATCGGTGGCTGA<br>plcBR: GGGTAGTCCGCTTTCGCTCTT	289	(Du et al., 2017)
iap	iapF: ACAAGCTGCACCTGTTGCAG<br>iapR: TGACAGCGTGTGTAGTAGCA	131	(Kaur et al., 2007)
