# Supplementary material for: Characterization of antibiogram fingerprints in Listeria monocytogenes recovered from irrigation water and agricultural soil samples
Source: PLoS One. 2020 Feb 10;15(2):e0228956. doi: 10.1371/journal.pone.0228956 (PMC7010277; doi:10.1371/journal.pone.0228956)
Supplement: S4 Table — (PDF) [file pone.0228956.s004.pdf]

S4 Table: The primer sequence and expected amplicon size used for the screening of resistance genes in *L. monocytogenes.*

Antibiotic class	Genes	PCR primer sequence (5′–3′)	PCR cycling condition	Amplicon size (bp)	Reference
Tetracyclines	*tet*A	F: GCTACATCCTGCTTGCCTTC<br>R: CATAGATCGCCGTGAAGAGG	94 °C,5m; 35[94 °C,1m; 55 °C,1m; 72 °C,1.5m]; 72 °C,5m	210	(Mendez et al., 1980)
	*tet*B	F: TTGGTTAGGGGCAAGTTTTG<br>R: GTAATGGGCCAATAACACCG	94 °C,5m; 35[94 °C,1m; 55 °C,1m; 72 °C,1.5m]; 72 °C,5m	659	(Marshall et al., 1983)
	*tet*C	F: CTTGAGAGCCTTCAACCCAG<br>R: ATGGTCGTCATCTACCTGCC	94 °C,5m; 35[94 °C,1m; 55 °C,1m; 72 °C,1.5m]; 72 °C,5m	418	(Marshall et al., 1983)
	*tet*D	F: AAACCATTACGGCATTCTGC<br>R: GACCGGATACACCATCCATC	94 °C,5m; 35[94 °C,1m; 55 °C,1m; 72 °C,1.5m]; 72 °C,5m	787	(Marshall et al., 1983)
	*tet*E	F: AAACCACATCCTCCATACGC<br>R: AAATAGGCCACAACCGTCAG	94 °C,5m; 35[94 °C,1m; 55 °C,1m; 72 °C,1.5m]; 72 °C,5m	278	(Marshall et al., 1986)
	*tet*G	F: GCTCGGTGGTATCTCTGCTC<br>R: AGCAACAGAATCGGGAACAC	94 °C,5m; 35[94 °C,1m; 55 °C,1m; 72 °C,1.5m]; 72 °C,5m	468	(Zhao and Aoki, 1992)
	*tet*K	F: TCGATAGGAACAGCAGTA<br>R: CAGCAGATCCTACTCCTT	94 °C,5m; 35[94 °C,1m; 55 °C,1m; 72 °C,1.5m]; 72 °C,5m	169	(Warsa et al., 1996)
	*tet*L	F: TCGTTAGCGTGCTGTCATTC<br>R: GTATCCCACCAATGTAGCCG	94 °C,5m; 35[94 °C,1m; 55 °C,1m; 72 °C,1.5m]; 72 °C,5m	267	(Burdett et al., 1982)
	*tet*M	F: GTGGACAAAGGTACAACGAG<br>R: CGGTAAAGTTCGTCACACAC	94 °C,5m; 35[94 °C,1m; 55 °C,1m; 72 °C,1.5m]; 72 °C,5m	406	(Warsa et al., 1996)
Sulfonamides	*sulI*	F: CGGCGTGGGCTACCTGAACG<br>R: GCCGATCGCGTGAAGTTCCG	94 °C,5m; 30[94 °C,30s; 65 °C,30s; 72 °C,2m]; 72 °C,10m	433	(Kerrn et al., 2002)

	sulII	F: GCGCTCAAGGCAGATGGCATT<br>R: GCGTTTGATACCGGCACCCGT	94 °C,5m; 30[94 °C,30s; 65 °C,30s; 72 °C,2m]; 72 °C,10m	293	(Kerrn et al., 2002)
Phenicols	*cmlA1*	F: CACCAATCATGACCAAG<br>R: GGCATCACTCGGCATGGACATG	94 °C,5m; 30[94 °C,30s; 50 °C,30s; 72 °C,1.5m]; 72 °C,5m	115	(Post and Hall, 2009)
	catI	F: AGTTGCTCAATGTACCTATAACC<br>R: TTGTAATTCATTAAGCATTCTGCC	94 °C,5m; 30[94 °C,30s; 50 °C,30s; 72 °C,1.5m]; 72 °C,5m	320	(Maynard et al., 2004)
	catII	F: ACACTTTGCCCTTTATCGTC<br>R: TGAAAGCCATCACATACTGC	94 °C,5m; 30[94 °C,30s; 50 °C,30s; 72 °C,1.5m]; 72 °C,5m	543	(Maynard et al., 2004)
Aminoglycosides	*strA*	F CTTGGTGATAACGGCAATTC<br>R: CCAATCGCAGATAGAAGGC	94 °C,4m; 30[94 °C,45s; 50 °C,45s; 72 °C,45s]; 72 °C,5m	348	(Velusamy et al., 2007)
	aadA	F: GTGGATGGCGGCCTGAAGCC<br>R: AATGCCCAGTCGGCAGCG	94 °C,4m; 30[94 °C,45s; 50 °C,45s; 72 °C,45s]; 72 °C,5m	525	(Velusamy et al., 2007)
	aac(3)-IIa (aacC2)[a]	F: CGGAAGGCAATAACGGAG<br>R: TCGAACAGGTAGCACTGAG	94 °C,5m; 30[94 °C,30s; 50 °C,30s; 72 °C,1.5m]; 72 °C,5m	428	(Maynard et al., 2004)
	aph(3)-Ia (aphA1)[a]	F: ATGGGCTCGCGATAATGTC<br>R: CTCACCGAGGCAGTTCCAT	94 °C,5m; 30[94 °C,30s; 50 °C,30s; 72 °C,1.5m]; 72 °C,5m	600	(Maynard et al., 2004)
	aph(3)-IIa (aphA2)[a]	F: GAACAAGATGGATTGCACGC<br>R: GCTCTTCAGCAATATCACGG	94 °C,5m; 30[94 °C,30s; 50 °C,30s; 72 °C,1.5m]; 72 °C,5m	510	(Maynard et al., 2004)
