# Supplementary material for: Characterization of antibiogram fingerprints in Listeria monocytogenes recovered from irrigation water and agricultural soil samples
Source: PLoS One. 2020 Feb 10;15(2):e0228956. doi: 10.1371/journal.pone.0228956 (PMC7010277; doi:10.1371/journal.pone.0228956)
Supplement: S5 Table — (PDF) [file pone.0228956.s005.pdf]

S5 Table: The primer sequence and expected amplicon size used for the screening of *AmpC* β-lactamase [65]and ESBLs in *L. monocytogenes* [43].

**PCR name**	**Targeted β-Lactamase(s)**	**Primer name**	**Primer sequence (5' –3')**	**Amplicon size (bp)**
Simplex AmpC	*AmpC*	AmpC_for<br>AmpC_rev	TTCTATCAAMACTGGCARCC<br>CCYTTTTATGTACCCAYGA	550
Multiplex I TEM, SHV and OXA-1-like	TEM variants including TEM-1 and TEM-2	MultiTSO-T_for<br>MultiTSO-T_rev	CATTTCCGTGTCGCCCTTATTC<br>CGTTCATCCATAGTTGCCTGAC	800
	SHV variants including SHV-1	MultiTSO-S_for<br>MultiTSO-S_rev	AGCCGCTTGAGCAAATTAAAC<br>ATCCCGCAGATAAATCACCAC	713
	OXA-1, OXA-4 and OXA-30	MultiTSO-O_for<br>MultiTSO-O_rev	GGCACCAGATTCAACTTTCAAG<br>GACCCCAAGTTTCCTGTAAGTG	564
Multiplex II CTX-M group 1, group 2 and group 9	Variants of CTX-M group 1 including CTX-M-1, CTX-M-3 and CTX-M-15	MultiCTXMGp1_for<br>MultiCTXMGp1-2_rev	TTAGGAARTGTGCCGCTGYA[b]<br>CGATATCGTTGGTGGTRCCAT[b]	688
	variants of CTX-M group 2 including CTXM-2	MultiCTXMGp2_for<br>MultiCTXMGp1-2_rev	CGTTAACGGCACGATGAC<br>CGATATCGTTGGTGGTRCCAT[b]	404
	Variants of CTX-M group 9 including CTX-M-9 and CTX-M-14	MultiCTXMGp9_for<br>MultiCTXMGp9_rev	TCAAGCCTGCCGATCTGGT<br>TGATTCTCGCCGCTGAAG	561
CTX-M group 8/25	CTX-M-8, CTX-M-25, CTX-M-26 and CTX-M-39 to CTX-M-41	CTX-Mg8/25_for<br>CTX-Mg8/25_rev	AACRCRCAGACGCTCTAC[b]<br>TCGAGCCGGAASGTGTYAT[b]	326
Multiplex III ACC, FOX, MOX, DHA, CIT and EBC (plasmid-mediated AmpC)	ACC-1 and ACC-2	MultiCaseACC_for<br>MultiCaseACC_rev	CACCTCCAGCGACTTGTTAC<br>GTTAGCCAGCATCACGATCC	346
	FOX-1 to FOX-5	MultiCaseFOX_for<br>MultiCaseFOX_rev	CTACAGTGCGGGTGGTTT<br>CTATTTGCGGCCAGGTGA	162
	MOX-1, MOX-2, CMY-1, CMY-8 to CMY-11 and CMY-19	MultiCaseMOX_for<br>MultiCaseMOX_rev	GCAACAACGACAATCCATCCT<br>GGGATAGGCGTAACTCTCCCAA	895
	DHA-1 and DHA-2	MultiCaseDHA_for	TGATGGCACAGCAGGATATTC	997

		MultiCaseDHA_rev	GCTTTGACTCTTTCGGTATTCG	
	LAT-1 to LAT-3, BIL-1, CMY-2 to MY-7, CMY-12 to CMY-18 and CMY-21 to CMY-23	MultiCaseCIT_for	CGAAGAGGCAATGACCAGAC	538
		MultiCaseCIT_rev	ACGGACAGGGTTAGGATAGY[b]	
	ACT-1 and MIR-1	MultiCaseEBC_for	CGGTAAAGCCGATGTTGCG	683
		MultiCaseEBC_rev	AGCCTAACCCCTGATACA	
Multiplex IV VEB, PER and GES	GES-1 to GES-9 and GES-11	MultiGES_for	AGTCGGCTAGACCGGAAAG	399
		MultiGES_rev	TTTGTCCGTGCTCAGGAT	
	PER-1 and PER-3	MultiPER_for	GCTCCGATAATGAAAGCGT	520
		MultiPER_rev	TTCGGCTTGACTCGGCTGA	
	VEB-1 to VEB-6	MultiVEB_for	CATTTCCCGATGCAAAGCGT	648
		MultiVEB_rev	CGAAGTTTCTTTGGACTCTG	
Multiplex V GES and OXA-48-like	GES-1 to GES-9 and GES-11	MultiGES_for	AGTCGGCTAGACCGGAAAG	399
		MultiGES_rev	TTTGTCCGTGCTCAGGAT	
	OXA-48-like	MultiOXA-48_for	GCTTGATCGCCCTCGATT	281
		MultiOXA-48_rev	GATTTGCTCCGTGGCCGAAA	
Multiplex VI IMP, VIM and KPC	IMP variants except IMP-9, IMP-16, IMP-18, IMP-22 and IMP-25	MultiIMP_for	TTGACACTCCATTTACDG[b]	139
		MultiIMP_rev	GATYGAGAATTAAGCCACYCT[b]	
	VIM variants including VIM-1 and VIM-2	MultiVIM_for[c]	GATGGTGTTTGGTCGCATA	390
		MultiVIM_rev[c]	CGAATGCGCAGCACCAG	
	KPC-1 to KPC-5	MultiKPC_for	CATTCAAGGGCTTTCTTGCTGC	538
		MultiKPC_rev	ACGACGGCATAGTCATTTGC	

[a]Annealing position within the corresponding open reading frame (from the base A of start codon ATG).

[b]Y=T or C; R=A or G; S=G or C; D=A or G or T.
