# Supplementary material for: Characterization of antibiogram fingerprints in Listeria monocytogenes recovered from irrigation water and agricultural soil samples
Source: PLoS One. 2020 Feb 10;15(2):e0228956. doi: 10.1371/journal.pone.0228956 (PMC7010277; doi:10.1371/journal.pone.0228956)
Supplement: S1 Fig — Lane 1 represents 100bp DNA ladder, lane 2 represents positive control (L. monocytogenes ATCC 9525), lane 3 represents negative control and lane 4 to lane 13 represents some of the positive isolates. (PDF) [file pone.0228956.s006.pdf]

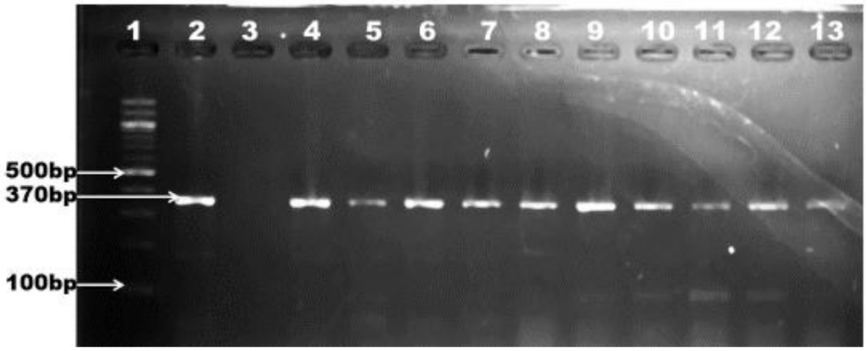

S1 Fig: Gel picture showing the molecular amplification of *prs* (370 bp) gene of *Listeria* spp. isolated from irrigation water and soil samples collected. Lane 1 represents 100bp DNA ladder, lane 2 represents positive control (*L. monocytogenes* ATCC 9525*)*, lane 3 represents negative control and lane 4 to lane 13 represents some of the positive isolates.
