# Supplementary material for: Characterization of antibiogram fingerprints in Listeria monocytogenes recovered from irrigation water and agricultural soil samples
Source: PLoS One. 2020 Feb 10;15(2):e0228956. doi: 10.1371/journal.pone.0228956 (PMC7010277; doi:10.1371/journal.pone.0228956)
Supplement: S1 Raw images — (PDF) [file pone.0228956.s008.pdf]

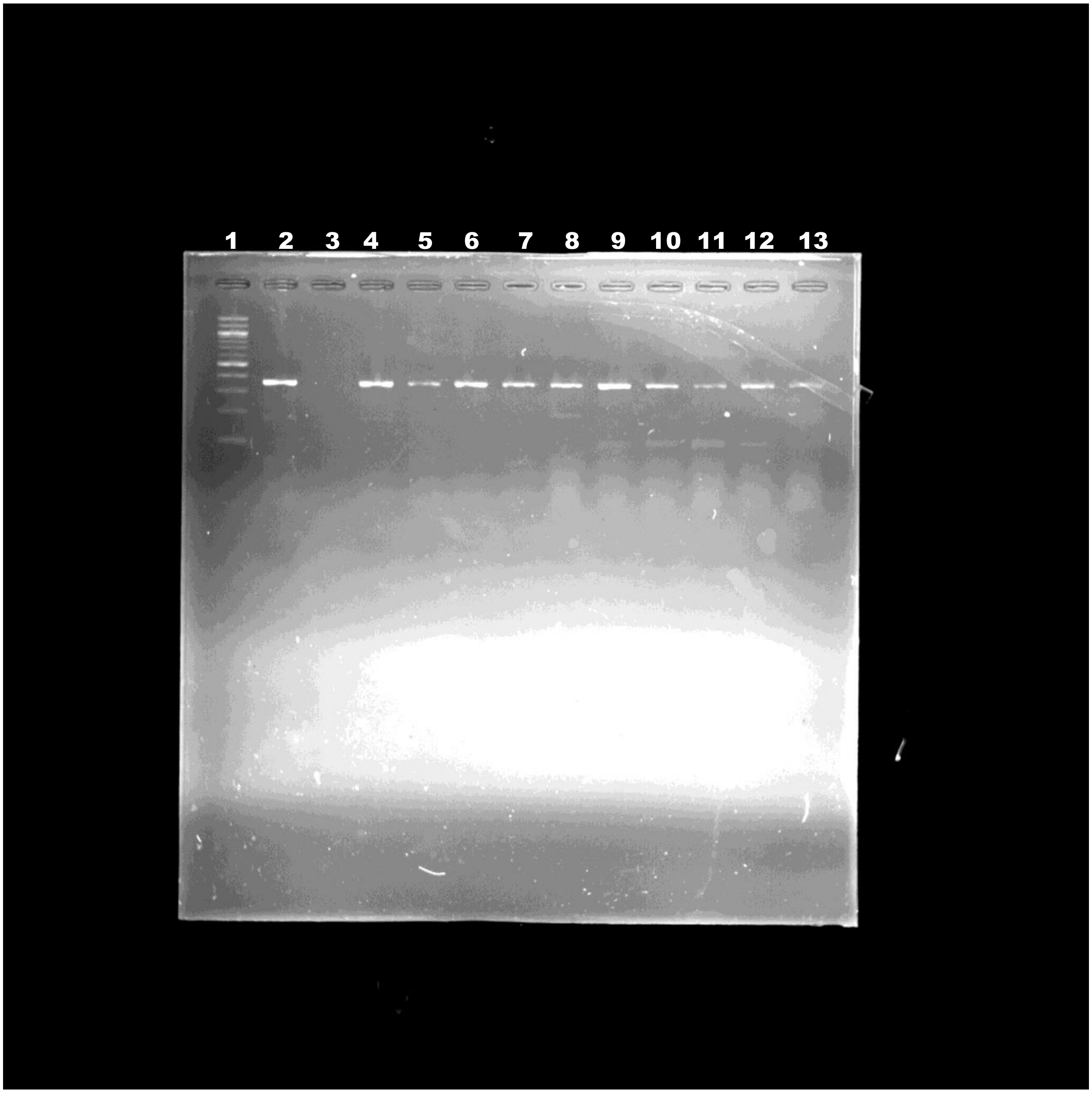

Raw gel picture showing the molecular amplification of *prs* (370 bp) gene of *Listeria* spp. isolated from irrigation water and soil samples collected. Lane 1 represents 100bp DNA ladder, lane 2 represents positive control (*L. monocytogenes* ATCC 9525*)*, lane 3 represents negative control and lane 4 to lane 13 represents some of the positive isolates.

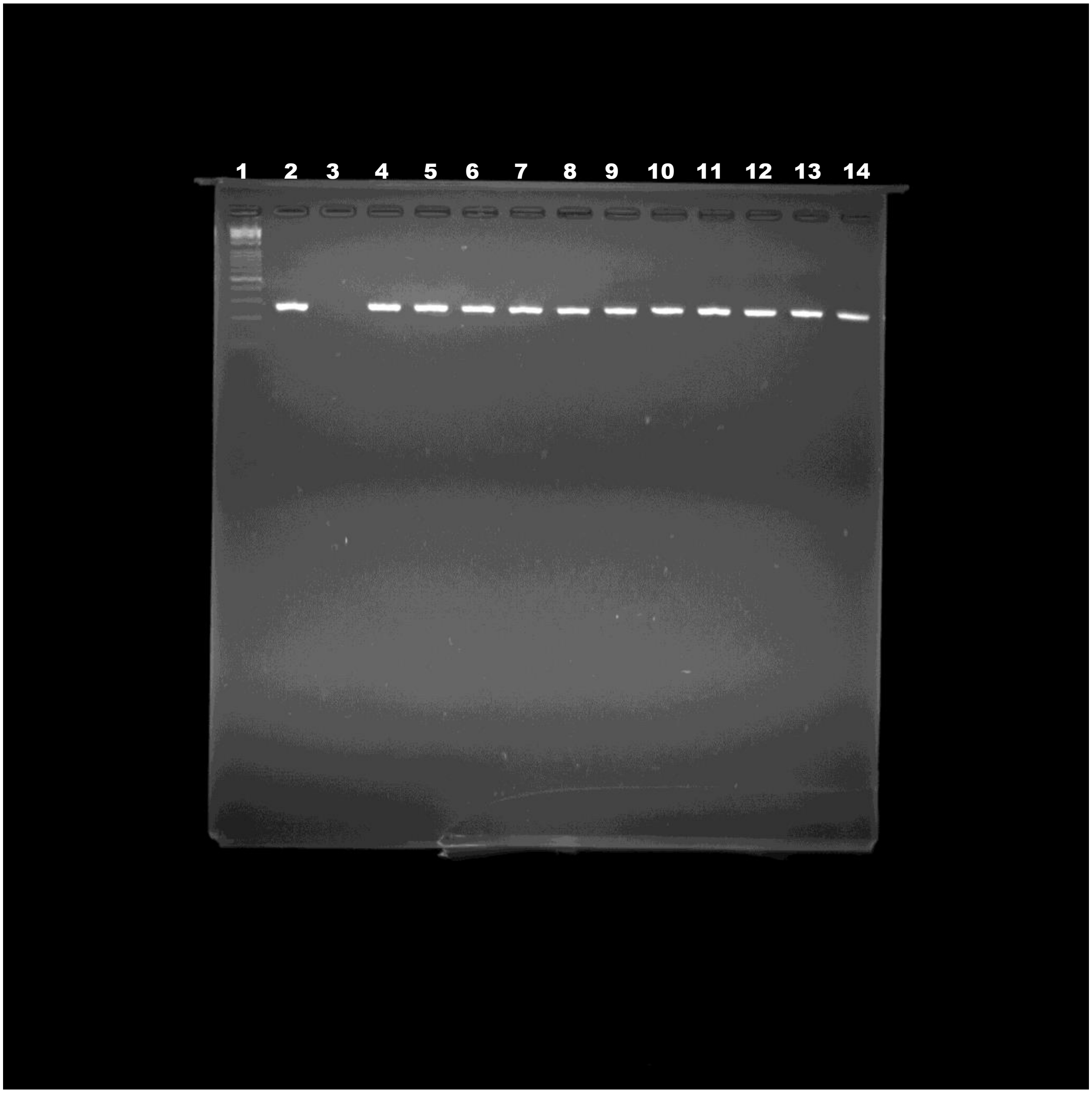

Raw gel picture showing the molecular amplification of *prfA* (274 bp) gene of *L. monocytogenes*. Lane 1 represents 100bp DNA ladder, lane 2 represents positive control (*L. monocytogenes* ATCC 9525*)*, lane 3 represents negative control and lane 4 to lane 14 represents some of the positive isolates.

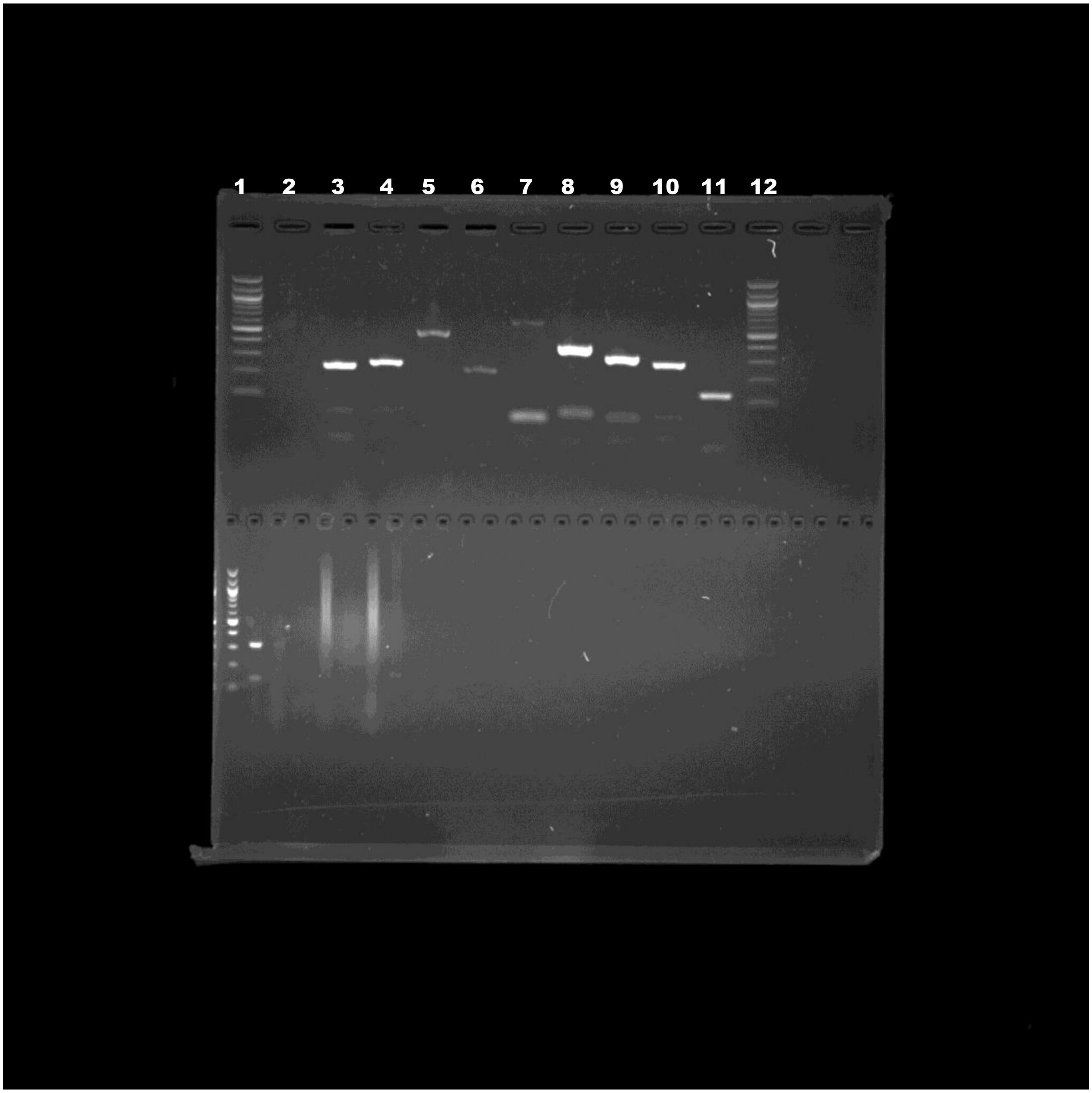

Raw gel picture showing multiplex PCR amplification of virulence genes; lane 3: *inlA* (256pb), lane 4: *inlB* (272bp), lane 5: *inlC* (517bp), lane 6: *inlJ* (238bp), lane 7: *actA* (650bp), lane 8: *hylA* (404bp), lane 9: *plcA* (326bp), lane 10: *plcB* (289bp), lane 11: *iap* (131bp), lane 1 and 12 represents 100bp DNA ladder and lane 2 represents negative control.
